# Synthesis and Biological Activity Screening of Newly Synthesized Trimethoxyphenyl-Based Analogues as Potential Anticancer Agents

**DOI:** 10.3390/molecules27144621

**Published:** 2022-07-20

**Authors:** Tarfah Al-Warhi, Matokah Abualnaja, Ola A. Abu Ali, Fayez Althobaiti, Fahad Alharthi, Fahmy G. Elsaid, Ali A. Shati, Eman Fayad, Doaa Elghareeb, Ali H. Abu Almaaty, Islam Zaki

**Affiliations:** 1 Department of Chemistry, College of Science, Princess Nourah Bint Abdulrahman University, Riyadh 11671, Saudi Arabia; tarfah-w@hotmail.com; 2 Department of Chemistry, Faculty of Applied Science, Umm Al-Qura University, Makkah Al Mukarrama 24381, Saudi Arabia; mmabualnaja@uqu.edu.sa; 3 Department of Chemistry, College of Science, Taif University, Taif 21944, Saudi Arabia; o.abuali@tu.edu.sa; 4 Department of Biotechnology, Faculty of Sciences, Taif University, Taif 21944, Saudi Arabia; faiz@tu.edu.sa (F.A.); e.esmail@tu.edu.sa (E.F.); 5 Department of Biology, College of Science, Taif University, Taif 21944, Saudi Arabia; f.alharthi@tu.edu.sa; 6 Biology Department, Science College, King Khalid University, Abha 61421, Saudi Arabia; felsaid@kku.edu.sa (F.G.E.); aaalshati@kku.edu.sa (A.A.S.); 7 Zoology Department, Faculty of Science, Mansoura University, Mansoura 35516, Egypt; 8 Department of Biology, Jumum College University, Umm Al-Qura University, Makkah 21955, Saudi Arabia; dekeshek@uqu.edu.sa; 9 Agriculture Genetic Engineering Research Institute (AGERI), Agriculture Research Centre, Cairo 12619, Egypt; 10 Zoology Department, Faculty of Science, Port Said University, Port Said 42526, Egypt; ali_zoology_2010@yahoo.com; 11 Pharmaceutical Organic Chemistry Department, Faculty of Pharmacy, Port Said University, Port Said 42526, Egypt

**Keywords:** diamide, oxazolone, imidazolone, triazinone, cytotoxicity, tubulin, cell cycle analysis, apoptosis

## Abstract

A group of novel trimethoxyphenyl (TMP)-based analogues were synthesized by varying the azalactone ring of 2-(3,4-dimethoxyphenyl)-4-(3,4,5-trimethoxybenzylidene)oxazolone **1** and characterized using NMR spectral data as well as elemental microanalyses. All synthesized compounds were screened for their cytotoxic activity utilizing the hepatocellular carcinoma (HepG2) cell line. Compounds **9**, **10** and **11** exhibited good cytotoxic potency with IC_50_ values ranging from 1.38 to 3.21 μM compared to podophyllotoxin (podo) as a reference compound. In addition, compounds **9**, **10** and **11** exhibited potent inhibition of β-tubulin polymerization. DNA flow cytometry analysis of compound **9** shows cell cycle disturbance at the G2/M phase and a significant increase in Annexin-V-positive cells compared with the untreated control. Compound **9** was further studied regarding its apoptotic potential in HepG2 cells; it decreased the level of MMP and Bcl-2 as well as boosted the level of p53 and Bax compared with the control HepG2 cells.

## 1. Introduction

Target-based chemotherapy is designed to exploit specific molecular targets within the body [1,2]. The ultimate goal of this type of therapy is to develop alternative and more effective drugs that specifically destroy the specific cellular target without having a significant effect on other cellular structures [3,4]. Microtubules are the most important validated target for the treatment of malignancies [5,6,7]. Microtubules, built from α,β-tubulin building units, are intimately involved in many diverse cellular structures as well as the replication of the cells and cell division [8,9]. During cell division, the microtubules required for mitosis begin to grow and form structures known as mitotic spindles [10]. These mitotic spindles begin to separate newly formed chromosomes to give new daughter cells [11]. Inhibition or decaying of the microtubule formation in tumor cells leads to the failure of formation of mitotic spindled and separation of chromosomes, which, eventually, inhibits cells’ reproduction and growth [12,13]. Thus, agents that interfere with the dynamics of tubulin may also act as inhibitors of cell division [14].

**Figure 1:**
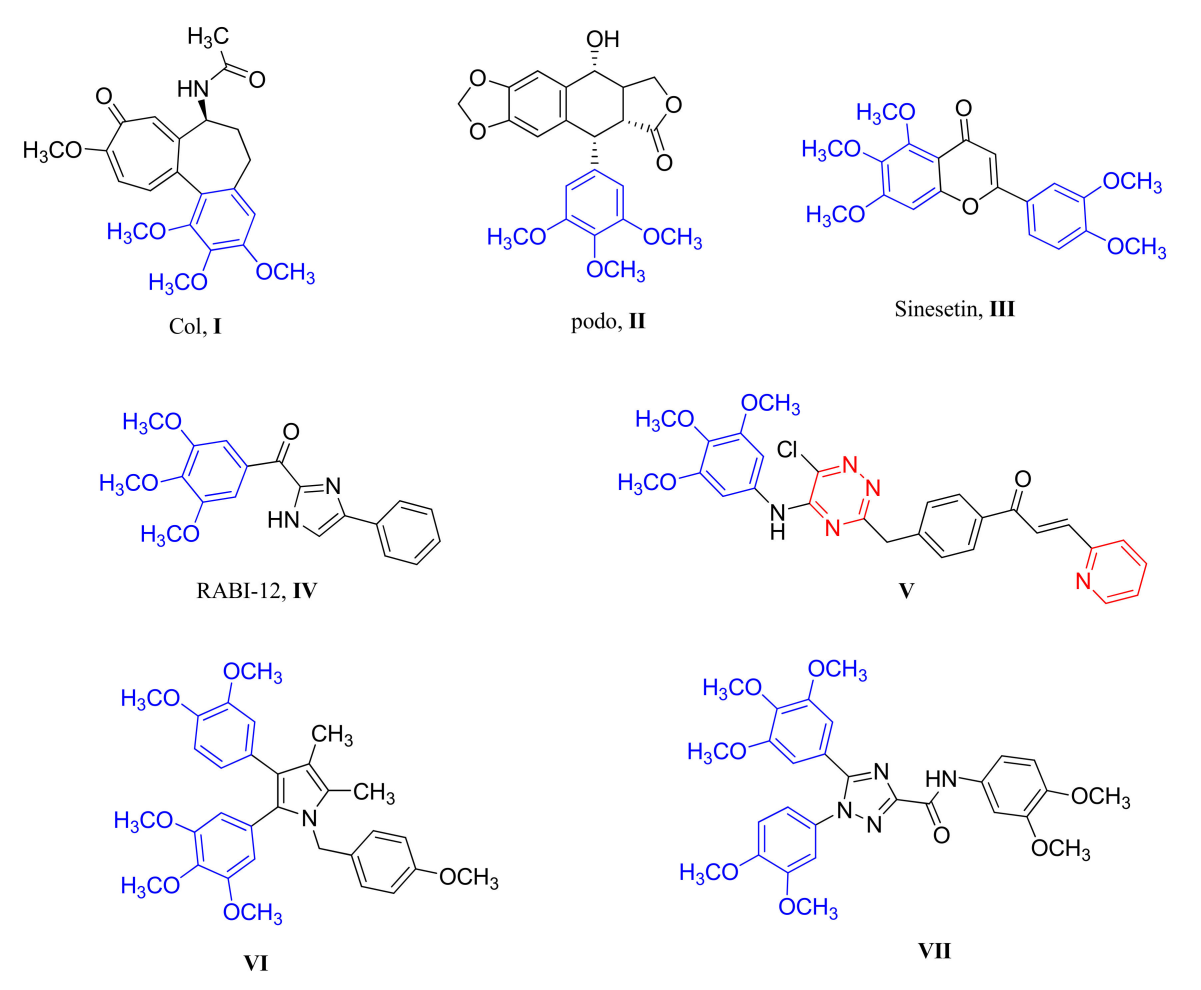
Chemical structure of col **I**, podo **II**, Sinensetin **III**, Imidazole **IV**, and 1,2,4-triazine **V** as anti-cancer agents and some reported tubulin polymerization inhibitors, **VI** and **VII**.

Many natural products possessing Trimethoxy Phenyl (TMP) rings, e.g., colchicine (col) **I** and podophyllotoxin (podo) **II**, were found to be potent cytotoxic agents that exert their cytotoxic properties by interfering with the tubulin-microtubule system [15,16]. Therefore, there has been considerable interest in the development of new anti-tubulin molecules based on these natural compounds as structural leads [17]. Sinensetin **III** is a plant-derived pentamethoxy flavone compound. It possesses potent cytotoxic properties toward a variety of cancer cell lines involving interaction with breast cancer resistance protein (ABCG2/BCRP) and antiangiogenesis [18] (Figure 1).

It was reported that diamide, imidazole, and 1,2,4-triazinone derivatives as a template for antiproliferative agents have cytotoxic activities over several cancer cell lines [19,20,21]. The average IC_50_ of imidazole RABI-112 **IV** over eight cancer cell lines was 14 nM, and it caused cells’ arrest at the G2/M phase of the cell cycle besides its detrimental effect on microtubule networks [22]. In addition, 1,2,4-triazine-chalcone hybrid **V** displayed significant antiproliferative activity against A-594, EC-109, PC-3, HCT-116, and MGC-803 cell lines with IC_50_ values of 0.52, 0.78, 0.61, 0.43, and 0.41 μM, respectively, compared with 5-fluorouracil (5-FU) as a reference control [23]. Additionally, compounds **VI** and **VII** contain dimethoxy phenyl (DMP) and TMP rings that exhibit cytotoxic potency over different cancer cell lines and promising tubulin polymerization inhibition activities [24,25] (Figure 1).

Based on the above structure analysis, the current study is concerned with the design and synthesis of new compounds decorated with DMP and TMP moieties starting from 2-(3,4-dimethoxyphenyl)-4-(3,4,5-trimethoxybenzylidene)oxazolone **1**. The prepared derivatives were subjected to in vitro cytotoxic evaluation utilizing a HepG2 cell line followed by a tubulin inhibition profile. Moreover, tubulin inhibition in HepG2 cells was found to trigger an apoptotic cascade. So that the apoptotic effects of the most potent compound were investigated through cell cycle analysis, inhibition of mitochondrial membrane potential, and determination of p53, Bax, and Bcl-2 levels.

## 2. Results and Discussion

### 2.1. Chemistry

**Scheme 1:**
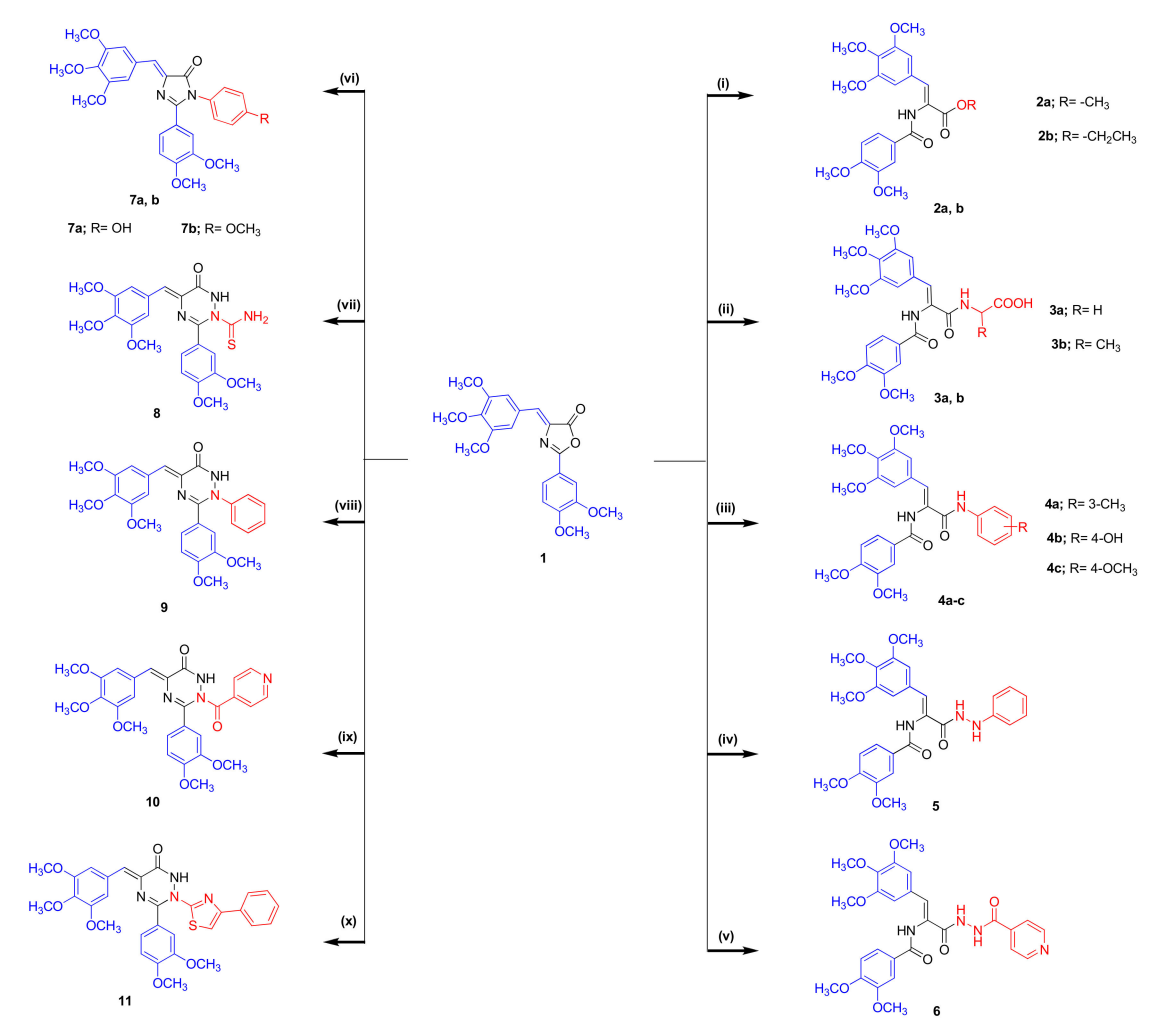
Synthesis of the target compounds **2**–**11**. Reagents and conditions: (**i**) Et_3_N, respective alcohol; (**ii**) respective amino acid, Et_3_N, EtOH; (**iii**) respective aryl amine, NaOAc, AcOH; (**iv**) phenyl hydrazine, EtOH; (**v**) isonicotinic acid hydrazide, EtOH; (**vi**) respective aryl amine, NaOAc, AcOH; (**vii**) thiosemicarbazide, NaOAc, AcOH; (**viii**) phenyl hydrazine, EtOH; (**ix**) isonicotinic acid hydrazide, NaOAc, AcOH; (**x**) 2-hydrazinyl-4-phenylthiazole, AcOH, DMF.

The synthetic strategy to prepare the target molecules is depicted in Scheme 1. The starting oxazolone was prepared in a good yield via reaction of 2-(3,4-dimethoxybenzamido)acetic acid with 3,4,5-trimethoxybenzaldehyde in acetic anhydride containing anhydrous sodium acetate according to a previously reported method [26]. Compound **1** was then subjected to a ring-opening reaction in refluxing methanol or absolute ethanol in the presence of triethyl amine (Et_3_N) as a base catalyst to provide methyl or ethyl acrylate ester **2a**,**b**, respectively. In addition, the interaction of oxazolone with respective amino acids, namely, glycine or alanine, in refluxing pure ethanol gave the corresponding carboxylic acid derivatives **4a** and **b**, respectively. Additionally, the synthesis of diamide molecules **4a**–**c** took place by heating to reflux a mixture of the key starting material **1** with appropriate aromatic amine, namely, *m*-toluidine, *p*-aminophenol, or *p*-anisidine, in glacial acetic acid for 1–2 h. Furthermore, compound **5** was obtained via reaction of the key starting material **1** with phenyl hydrazine at room temperature for 3 h. Moreover, refluxing the oxazolone **1** with isonicotinic acid hydrazide in pure ethanol resulted in the formation of pyridine carbohydrazide derivative **6**. On the other hand, cyclocondensation of the oxazolone **1** with respective aryl amine in glacial acetic acid in the presence of anhydrous sodium acetate furnished the corresponding *N*-aryl imidazolone derivatives **7a** and **b**. The preparation of *N*-thioamide-1,2,4-triazinone derivative **8** was achieved by the reaction of oxazolone **1** with thiosemicarbazide in glacial acetic acid in the presence of anhydrous sodium acetate, while its reaction with phenyl hydrazine under reflux yielded the *N*-phenyl-1,2,4-triazinone molecule **9**. Moreover, the *N*-pyridoyl-1,2,4-triazinone derivative **10** was prepared by condensing oxazolone **1** with isonicotinic acid hydrazide in refluxing glacial acetic acid in the presence of anhydrous sodium acetate. Finally, *N*-phenylthiazole-1,2,4-triazinone **11** was achieved via the reaction of the key starting material **1** with 2-hydrazinyl-4-phenylthiazole in dry DMF containing a few drops of glacial acetic acid.

### 2.2. Biology

#### 2.2.1. Cytotoxic Activity against Liver HepG2 Cell Line

All the newly synthesized analogs were evaluated for their in vitro cytotoxicity against the hepatocellular carcinoma HepG2 cell line. Podo was utilized as a reference control in the current study. The results over HepG2 cells revealed that compounds **9**, **10**, and **11** exhibited potent cytotoxic activity with IC_50_ ranges within 1.38–3.21 μM. The *N*-phenyl triazinone derivative **9** (IC_50_ = 1.38 μM) has proved to be the most potent followed by the *N*-pyridoyl triazinone **10** (IC_50_ = 2.52 μM) and the *N*-phenylthiazolyl triazinone **11** (IC_50_ = 3.21 μM). In addition, compounds **3a**, **5**, **6**, **7a**, **7b**, and **8** displayed moderate cytotoxic activity with an IC_50_ concentration of less than 10 μM. Compounds **2a**, **2b**, **3b**, and **4b** showed relatively weak cytotoxic activity with an IC_50_ concentration of less than 20 μM. The *N*-phenyl triazinone derivative **9** was further investigated for its cytotoxic effects against normal liver cell line HL-7702. The results showed a selective mode of cytotoxic effect with an IC_50_ value of 29.07 μM. These results indicate the selectivity of *N*-phenyl triazinone **9** for tumor HepG2 cells and its selectivity for the normal liver cell line (Table 1).

#### 2.2.2. Tubulin Polymerization Inhibition Activity

**Figure 2:**
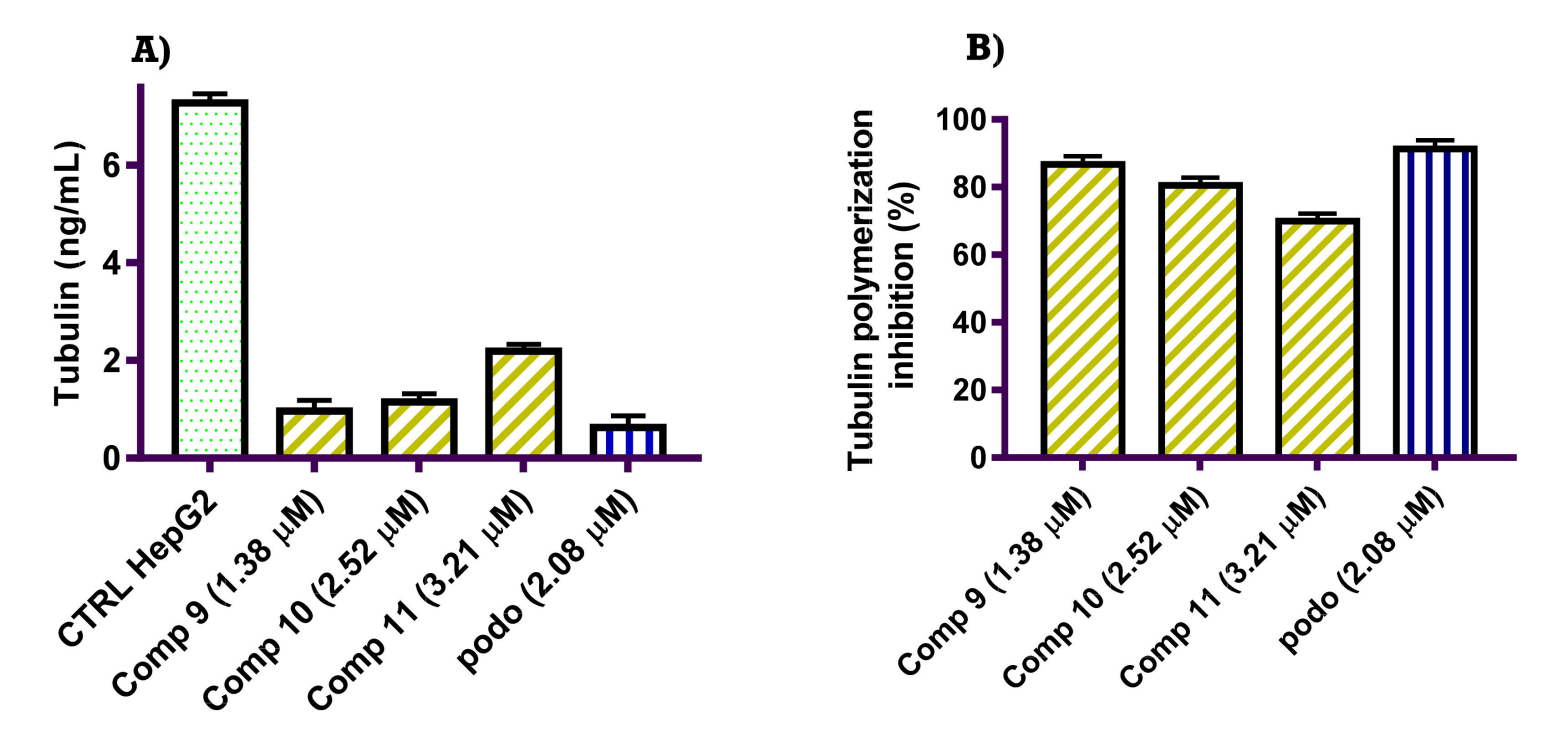
Compounds **9**, **10**, and **11** inhibited β-tubulin in HepG2 cells. (**A**) HepG2 cells were treated with 1.38, 2.52, 3.21, and 2.08 μM of compounds **9**, **10**, **11**, and podo, respectively, and tubulin percentage was determined by ELISA assay. (**B**) β-Tubulin polymerization inhibition percentage induced by compounds **9**, **10**, and **11** compared to podo at their IC_50_ concentration (μM).

To confirm whether the observed cytotoxic potency of our designed compounds was correlated to β-tubulin inhibition activity, the inhibitory effect of the most cytotoxic compounds **9**, **10**, and **11** on the tubulin polymerization was carried out using podo as a positive control. The selected candidates were tested at a concentration equal to their IC_50_ concentration dose value. ELISA analysis results revealed that compounds **9** and **10** displayed potent β-tubulin polymerization inhibition with percent inhibition of 86.73% and 80.51%, respectively, compared to podo (91.32% β-tubulin polymerization inhibition). On the other hand, β-tubulin polymerization inhibition percentage for compound **11** was recorded as 69.95%. It could be assumed from these results that the molecular target of these analogs is indeed tubulin (Figure 2).

#### 2.2.3. Cell Cycle Analysis of Compound **9**

**Figure 3:**
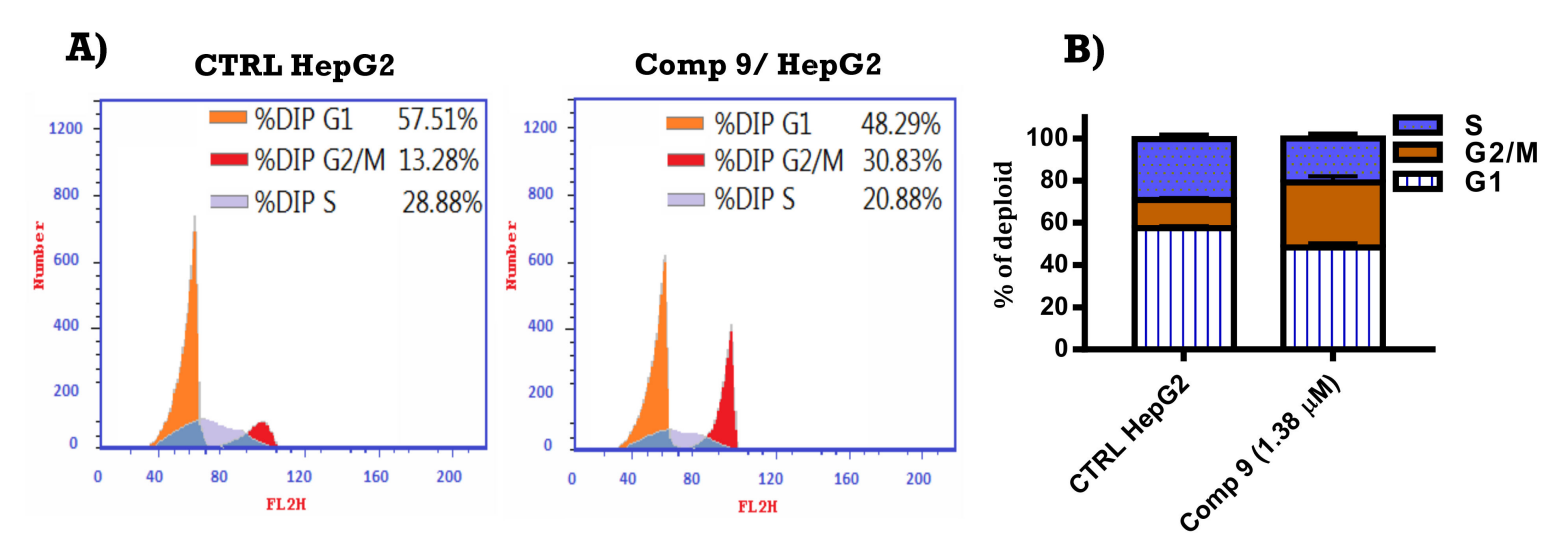
Compound **9** induces G2/M phase disturbance in HepG2 cells. (**A**) HepG2 cells were treated with 1.38 μM of compound **9** for 48 h. Cell apoptosis was quantized by PI and FACS analysis using image-based cytometry. (**B**) The percentage of cells in different phases was quantized.

It is well documented that exposure to microtubule targeting agents leads to the malformed mitotic spindle which eventually leads to cell cycle arrest at the G2/M phase and apoptosis induction [27]. Thus, the effect of the selected candidate **9** on the cell cycle progression of the HepG2 cell line was examined by flow cytometry analysis (FACS). The HepG2 cell line was treated with the IC_50_ concentration of compound **9** for 48 h. The FACS analysis results reveal that the selected compound **9** resulted in G2/M phase arrest as denoted by the increase in the G2/M cell population percentage (30.83%) compared with the control sample (13.28%) with about a 3-fold increase compared to the untreated control sample. In conclusion, compound **9** showed HepG2 inhibition of cell growth via cell cycle arrest at the G2/M phase (Figure 3).

#### 2.2.4. Apoptosis-Inducing Activity of Compound **9**

**Figure 4:**
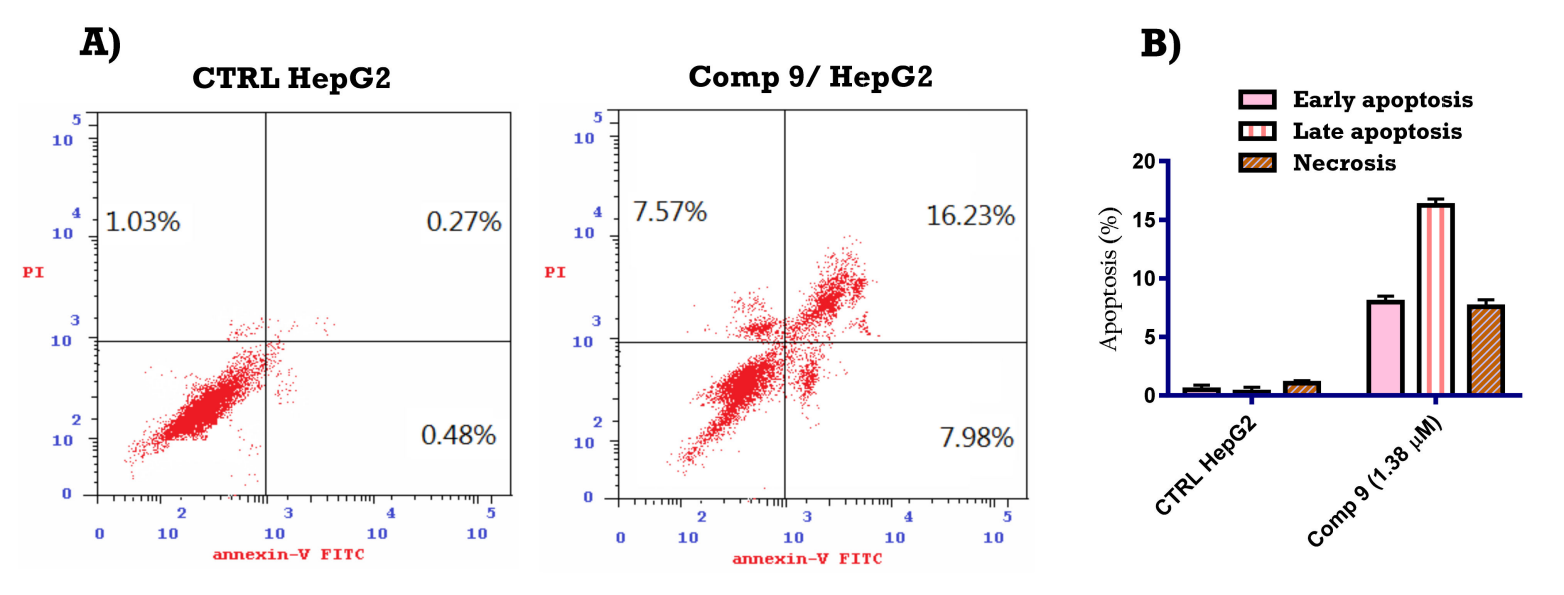
Compound **9** induces apoptosis in HepG2 cells. (**A**) HepG2 cells were treated with 1.38 μM of compound **9** for 48 h. Cell apoptosis was quantized by Annexin V-FITC/PI dual staining assay using image-based cytometry. (**B**) The quantification of HepG2 cell apoptosis.

The extent of apoptosis induced by compound **9** in HepG2 cells was quantified by the FACS analysis. Cells were harvested after 48 h and analyzed for apoptosis percentage by flow cytometry. Figure 4 showed the percentage of cells in each stage of apoptosis induced by compound **9** at a concentration equal to its IC_50_ dose value compared with the untreated sample. The results obtained for compound **9** showed a large increase in cells in the early and late stages after 48 h of exposure. It could be noticed that compound **9** increases the percentage of early and late apoptosis by 16.63- and 60.11-fold, respectively, compared with the untreated control. These results indicate that the compound’s mechanism of action may occur via the apoptosis pathway mechanism.

#### 2.2.5. Mitochondrial Membrane Potential

**Figure 5:**
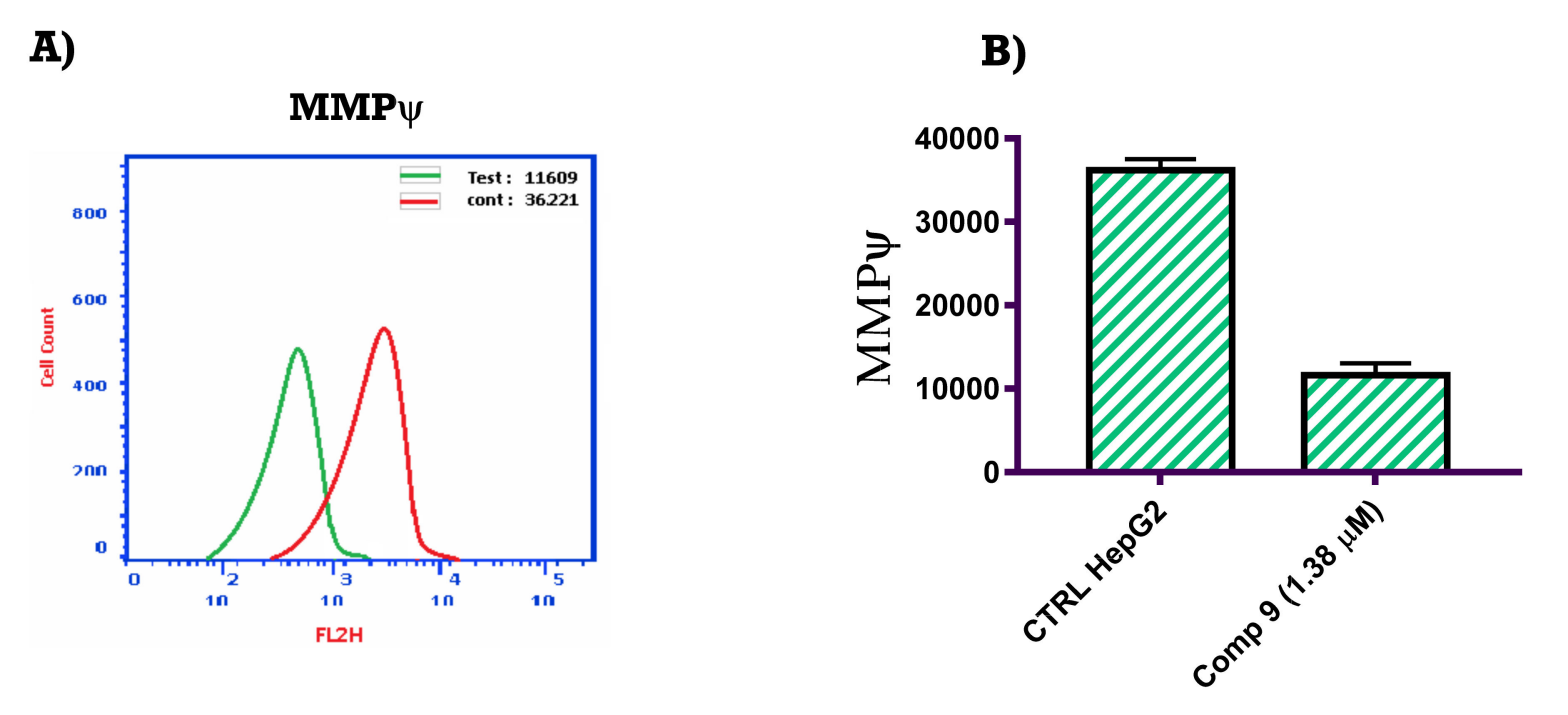
Compound **9** promotes MMP dissipation in HepG2 cells. (**A**) HepG2 cells were treated with 1.38 μM of compound **9** for 48 h. MMP was quantized using image-based flow cytometry. (**B**) The quantification of MMP in HepG2 cells after treatment with compound **9** at its IC_50_ concentration (μM).

Mitochondrial Membrane Potential (MMP) plays a vital role in the initiation of the apoptosis process [28]. Loss of MMP is characteristic of an early stage of apoptosis [29]. In the current study, HepG2 cells were treated with compound **9** at a concentration level equal to its IC_50_ concentration (1.38 μM), and MMP was determined using FACS analysis. The results indicate that compound **9** significantly reduced MMP compared with the untreated control HepG2 cells. From Figure 5, it is noticeable that compound **9** decreases the level of MMP by 3.12-fold compared with negative HepG2 cells. These results reveal that compound **9** exerted its cytotoxic activity by promoting MMP dissipation in the G2/M phase in HepG2 cells.

#### 2.2.6. In Vitro ELISA Quantification of the Level of p53, Bax and Bcl-2

**Figure 6:**
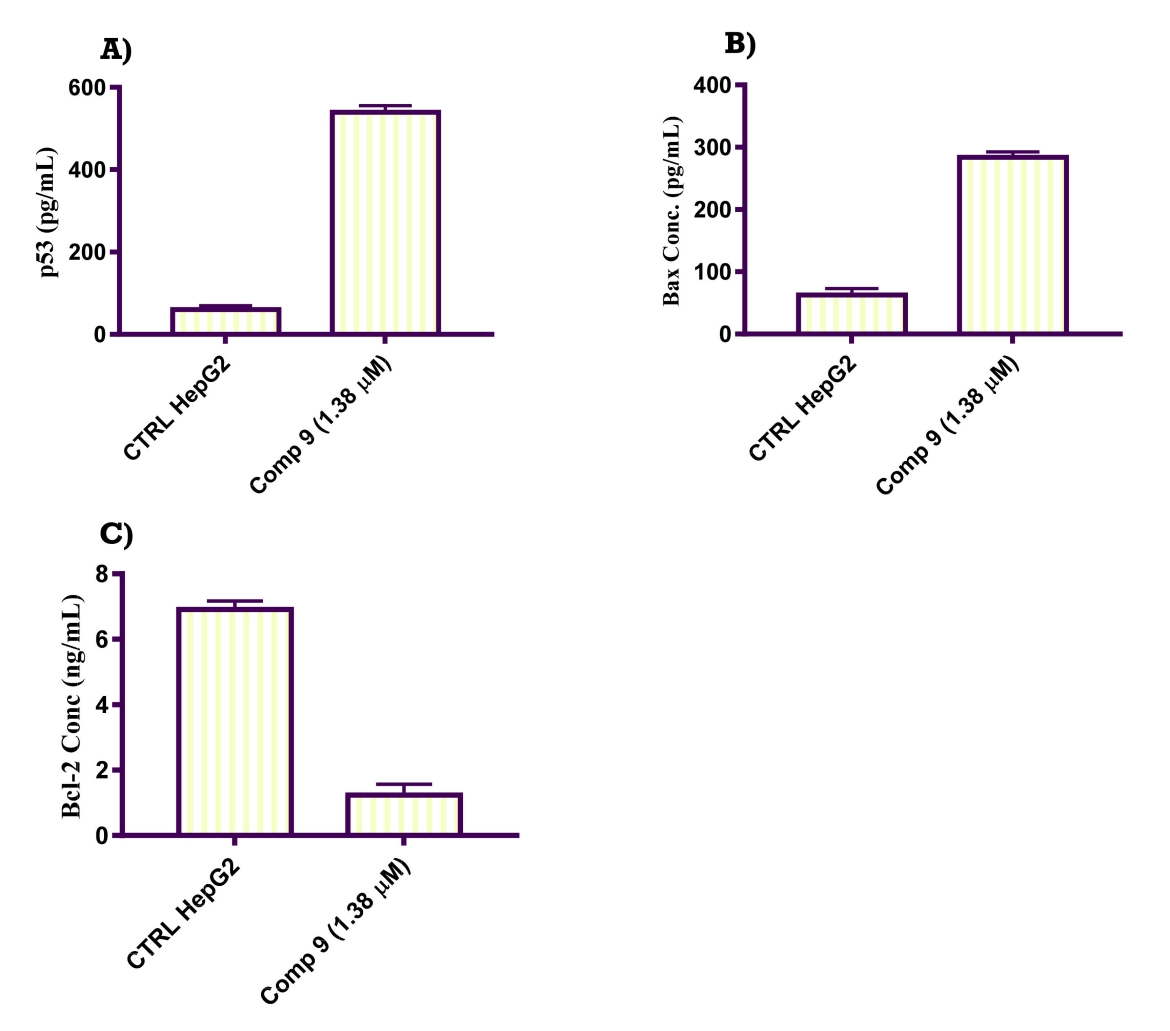
(**A**) The quantification of p53. (**B**) The quantification of Bax. (**C**) The quantification of Bcl-2. HepG2 cells were treated with 1.38 μM of compound **9** for 48 h and apoptotic markers were determined by ELISA assay.

Many studies suggested that tubulin-inhibiting agents cause G2/M phase arrest followed by apoptosis induction [30]. Compound **9** was screened for its ability to activate p53 and Bax as well as downregulate the level of Bcl-2 to investigate the mechanistic apoptotic pathway that catalyzed the apoptosis process [30]. HepG2 cells were treated with compound **9** at a concentration equal to its IC_50_ in μM, and the level of p53, Bax, and Bcl-2 was quantized using ELISA analysis. The results obtained for compound **9** show the increase in the level of p53 and Bax by 8.91- and 4.50-fold, respectively, compared with the negative control samples. Similarly, compound **9** showed a decrease in the level of Bcl-2 by 5.58-fold less than the negative control samples. The results indicate that compound **9**′s mechanisms of action induce the intrinsic pathway of the apoptotic mechanism through increasing the level of p53 and Bax, together with decreasing the level of Bcl-2 (Figure 6).

#### 2.2.7. Molecular Docking Study

**Figure 7:**
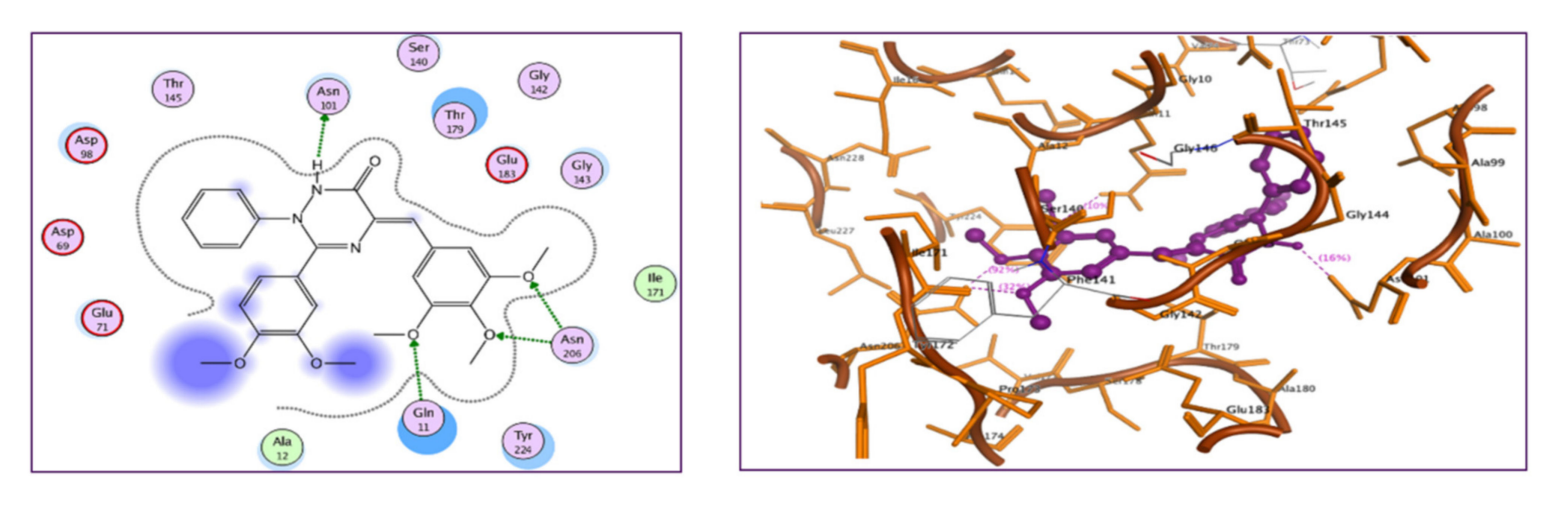
The binding poses (2D and 3D) of *N*-phenyl triazinone **9** in tubulin active site (PDB ID: 5LYJ).

*N*-phenyl triazinone **9** was docked into the validated binding site of the tubulin crystal structure (PDB code: 5LYJ). The *N*-phenyl triazinone ligand interacted with the active site of 5LYJ by four hydrogen bonds. *N*-phenyl triazinone molecule **9** interacted with its NH group of triazinone rings as a hydrogen bond donor with amino acid Asn 101 residue. Additionally, *N*-phenyl triazinone molecule **9** interacted with its TMP ring as a hydrogen bond acceptor by three hydrogen bonds with amino acids Gln 11 and Asn 206. Furthermore, the docking score achieved by *N*-phenyl triazinone molecule **9** was −14.67 kcal/mol (Figure 7). In conclusion, the interaction results are consistent with the obtained results of tubulin polymerization inhibitory activity and justify the observed experimental findings.

## 3. Experimental

### 3.1. Chemistry: General

Melting points were determined in open capillary tube using Electrothermal Digital melting point apparatus and were uncorrected. ^1^H-NMR and ^13^C-NMR spectra were obtained with a Bruker 400 MHz DRX-Avance NMR spectrometer, and peak positions are given in ppm downfield from tetramethylsilane (TMS) as the internal standard. Elemental analyses were performed on Elementar, Vario El, Microanalytical unit, Cairo, Egypt, and were found within ±0.4% of the theoretical values. Chemicals and reagents were obtained from commercial sources and used without further purification.

### 3.2. Chemistry

#### 3.2.1. General Procedure for the Preparation of (*Z*)-2-(3,4-Dimethoxyphenyl)-4-(3,4,5-Trimethoxybenzylidene)oxazol-5(4*H*)-one (**1**)

A mixture of 3,4,5-trimethoxybenzaldehyde (2.39 g, 10 mmol), 2-(3,4-dimethoxy benzamido)acetic acid (1.96 g, 10 mmol) and anhydrous sodium acetate (0.90 g, 11 mmol) in acetic anhydride (10 mL) was heated in oil bath at 80 °C for 2 h. After heating time, 100 mL of absolute ethanol was added to the reaction mixture, and the mixture was allowed to stand overnight. The precipitate formed was filtered off, washed with two 25 mL portions of ice-cold ethanol, dried and crystallized from ethanol/water (3:1) to get pure compound **1**.

Yellow powder (2.48 g, 54.33%), m.p. 195–197 °C. ^1^H-NMR (400 MHz, DMSO-*d_6_*, ppm): 3.76 (s, 3*H*, OCH_3_), 3.85 (s, 3*H*, OCH_3_), 3.87 (s, 6*H*, 2OCH_3_), 3.88 (s, 3*H*, OCH_3_), 7.21–7.16 (m, 2*H*, arom.CH and olefinic CH), 7.56 (d, *J* = 1.6 Hz, 1*H*, arom.CH), 7.67 (dd, *J* = 8.4, 1.7 Hz, 1*H*, arom.CH), 7.74 (s, 2*H*, arom.CH). ^13^C-NMR (100 MHz, DMSO-*d_6_*, ppm): 55.34 (OCH_3_), 55.76 (2OCH_3_), 55.89 (OCH_3_), 60.25 (OCH_3_), 109.67 (C2,6 trimethoxybenzylidene), 111.95 (C5 dimethoxyphenyl), 117.00 (C2 dimethoxyphenyl), 122.18 (C6 dimethoxyphenyl), 128.97 (C olefinic), 129.43 (C1 dimethoxyphenyl), 131.25 (C4 oxazolone), 132.17 (C1 trimethoxybenzylidene), 140.14 (C4 trimethoxybenzylidene), 149.00 (C3 dimethoxyphenyl), 152.80 (C4 dimethoxyphenyl), 153.51 (C3,5 trimethoxybenzylidene), 162.37 (C2 oxazolone), 167.04 (C=O oxazolone). Anal. Calcd. for C_21_H_21_NO_7_ (399.39): C, 63.15; H, 5.30; N, 3.51. Found: C, 63.08; H, 5.36; N, 3.54.

##### 3.2.2. General Procedure for the Preparation of Ester Derivatives **2a**,**b**

A suspension of 2-(3,4-dimethoxyphenyl)-4-(3,4,5-trimethoxybenzylidene)oxazolone **1** (399 mg, 1 mmol) in respective alkyl alcohol (25 mL), namely, methyl alcohol or ethyl alcohol, was added to Et_3_N (0.5 mL). The resulting mixture was heated to reflux for 4–5 h until complete on thin-layer chromatography. Evaporation of the solvent afforded the crude ester product that was crystallized from appropriate solvent to get pure compound **2a**,**b**.

##### (*Z*)-Methyl 2-(3,4-Dimethoxybenzamido)-3-(3,4,5-Trimethoxyphenyl)acrylate (**2a**)

White powder (273 mg, 63.41%), crystallized from methanol/H_2_O (3:1), m.p. 248–250 °C. ^1^H-NMR (400 MHz, DMSO-*d_6_*, δ ppm): 3.66 (s, 6*H*, 2OCH_3_), 3.66 (s, 3*H*, OCH_3_), 3.73 (s, 3*H*, OCH_3_), 3.80 (s, 3*H*, OCH_3_), 3.83 (s, 3*H*, OCH_3_), 7.05–7.12 (m, 3*H*, arom.CH and olefinic CH), 7.39 (s, 1*H*, arom.CH), 7.60 (s, 1*H*, arom.CH), 7.67 (dd, *J* = 8.4, 1.7 Hz, 1*H*, arom.CH), 9.93 (s, 1*H*, NH). ^13^C-NMR (100 MHz, DMSO-*d_6_*, ppm): 52.22 (OCH_3_), 55.62 (OCH_3_), 55.67 (OCH_3_), 55.71 (OCH_3_), 60.11 (2OCH_3_), 107.67 (C2,6 trimethoxyphenyl), 110.72 (C5 dimethoxybenzamide), 111.00 (C2 dimethoxybenzamide), 121.18 (C6 dimethoxybenzamide), 125.29 (C olefinic), 125.84 (C1 trimethoxyphenyl), 128.79 (C1 dimethoxybenzamide), 133.27 (C olefinic), 138.69 (C4 trimethoxyphenyl), 148.35 (C3 dimethoxybenzamide), 151.86 (C4 dimethoxybenzamide), 152.62 (C3,5 trimethoxyphenyl), 165.36 (C=O amide), 165.68 (C=O dimethoxybenzamide). Anal. Calcd. for C_22_H_25_NO_8_ (431.44): C, 61.25; H, 5.84; N, 3.25. Found: C, 61.29; H, 5.91; N, 3.18.

##### (*Z*)-Ethyl 2-(3,4-Dimethoxybenzamido)-3-(3,4,5-Trimethoxyphenyl)acrylate (**2b**)

White powder (267 mg, 59.92%), crystallized from ethanol/H_2_O (3:1), m.p. 248–250 °C. ^1^H-NMR (400 MHz, DMSO-*d_6_*, δ ppm): 1.25 (t, J = 7.1 Hz, 3*H*, OCH_2_CH_3_), 3.67 (s, 6*H*, 2OCH_3_), 3.67 (s, 3*H*, OCH_3_), 3.81 (s, 3*H*, OCH_3_), 3.84 (s, 3*H*, OCH_3_), 4.20 (q, J = 7.1 Hz, 2*H*, OCH_2_CH_3_), 7.08 (s, 2*H*, arom.CH), 7.10 (s, 1*H*, olefinic CH), 7.38 (s, 1*H*, arom.CH), 7.60 (s, 1*H*, arom.CH), 7.64–7.70 (m, 1*H*, arom.CH), 9.92 (s, 1*H*, NH). ^13^C-NMR (100 MHz, DMSO-*d_6_*, δ ppm): 14.19 (OCH_2_CH_3_), 55.60 (OCH_3_), 55.65 (OCH_3_), 55.70 (2OCH_3_), 60.09 (OCH_3_), 60.80 (OCH_2_CH_3_), 107.62 (C2,6 trimethoxyphenyl), 110.71 (C5 dimethoxybenzamide), 111.00 (C2 dimethoxybenzamide), 121.13 (C6 dimethoxybenzamide), 125.42 (C olefinic), 126.16 (C1 trimethoxyphenyl), 128.84 (C1 dimethoxybenzamide), 132.96 (C olefinic), 138.61 (C4 trimethoxyphenyl), 148.33 (C3 dimethoxybenzamide), 151.82 (C4 dimethoxybenzamide), 152.60 (C3,5 trimethoxyphenyl), 165.09 (C=O amide), 165.43 (C=O dimethoxybenzamide). Anal. Calcd. for C_23_H_27_NO_8_ (445.46): C, 62.01; H, 6.11; N, 3.14. Found: C, 61.95; H, 6.17; N, 3.06.

#### 3.2.3. General Procedure for the Preparation of 2-(2-(3,4-Dimethoxybenzamido)-3-(3,4,5-Trimethoxyphenyl)acrylamido)carboxylic Acids **3a**,**b**

To a suspension of 2-(3,4-dimethoxyphenyl)-4-(3,4,5-trimethoxybenzylidene)oxazolone **1** (399 mg, 1 mmol) in absolute ethanol, we added respective amino acid (1 mmol), Et_3_N (10 drops), and the reaction mixture was heated to reflux for 10 h. After completion of the reaction, water was added (100 mL) and the organic layer was extracted with EtOAc. The organic layer was evaporated under reduced pressure. The crude product was purified by crystallization from ethanol/H2O (3:1) to afford pure compound **3a**,**b**.

##### (*Z*)-2-(2-(3,4-Dimethoxybenzamido)-3-(3,4,5-Trimethoxyphenyl)acrylamido)acetic Acid (**3a**)

White powder (238 mg, 50.17%), m.p. 248–250 °C. ^1^H-NMR (400 MHz, DMSO-*d_6_*, δ ppm): 3.44 (s, 2*H*, CH_2_), 3.59 (s, 6*H*, 2OCH_3_), 3.63 (s, 3*H*, OCH_3_), 3.80 (s, 3*H*, OCH_3_), 3.83 (s, 3*H*, OCH_3_), 6.94 (s, 2*H*, arom.CH), 7.08 (d, *J* = 8.1 Hz, 1*H*, arom.CH), 7.34 (s, 1*H*, olefinic CH), 7.49 (s, 1*H*, NH), 7.63 (s, 1*H*, arom.CH), 7.69 (d, *J* = 8.4 Hz, 1*H*, arom.CH), 9.95 (s, 1*H*, NH). ^13^C-NMR (100 MHz, DMSO-*d_6_*, δ ppm): 44.19 (CH_2_), 55.59 (2OCH_3_), 55.63 (OCH_3_), 55.68 (OCH_3_), 60.05 (OCH_3_), 107.03 (C2,6 trimethoxyphenyl), 110.92 (C5 dimethoxybenzamide), 111.04 (C2 dimethoxybenzamide), 121.36 (C6 dimethoxybenzamide), 125.58 (C olefinic), 129.21 (C1 trimethoxyphenyl), 129.59 (C1 dimethoxybenzamide), 129.87 (C olefinic), 137.94 (C4 trimethoxyphenyl), 148.28 (C3 dimethoxybenzamide), 151.82 (C4 dimethoxybenzamide), 152.56 (C3,5 trimethoxyphenyl), 163.96 (C=O amide), 165.50 (C=O dimethoxybenzamide), 172.01 (C=O carboxylic). Anal. Calcd. for C_23_H_26_N_2_O_9_ (474.46): C, 58.22; H, 5.52; N, 5.90. Found: C, 58.29; H, 5.54; N, 5.84.

##### (*Z*)-2-(2-(3,4-Dimethoxybenzamido)-3-(3,4,5-Trimethoxyphenyl)acrylamido)propanoic Acid (**3b**)

White powder (236 mg, 48.22%), m.p. 248–250 °C. ^1^H-NMR (400 MHz, DMSO-*d_6_*, δ ppm): 1.33 (d, *J* = 7.2 Hz, 3*H*. CH_3_), 3.60 (s, 6*H*, 2OCH_3_), 3.64 (s, 3*H*, OCH_3_), 3.80 (s, 3*H*, OCH_3_), 3.83 (s, 3*H*, OCH_3_), 4.34 (p, *J* = 7.3 Hz, 1*H*, CH), 6.94 (s, 2*H*, arom.CH), 7.07 (d, *J* = 8.4 Hz, 1*H*, arom.CH), 7.25 (s, 1*H*), 7.63 (s, 1*H*, arom.CH), 7.69 (d, *J* = 8.2 Hz, 1*H*, arom.CH), 8.21 (d, *J* = 7.3 Hz, 1*H*, NH), 9.73 (s, 1*H*, NH), 12.38 (s, 1*H*, OH). ^13^C-NMR (100 MHz, DMSO-*d_6_*, δ ppm): 17.28 (CH_3_), 48.19 (CH), 55.68 (2OCH_3_), 55.72 (OCH_3_), 55.76 (OCH_3_), 60.15 (OCH_3_), 107.09 (C2,6 trimethoxyphenyl), 110.93 (C5 dimethoxybenzamide), 111.17 (C2 dimethoxybenzamide), 121.48 (C6 dimethoxybenzamide), 125.87 (C olefinic), 129.08 (C1 trimethoxyphenyl), 129.66 (C1 dimethoxybenzamide), 129.91 (C olefinic), 138.03 (C4 trimethoxyphenyl), 148.29 (C3 dimethoxybenzamide), 151.83 (C4 dimethoxybenzamide), 152.64 (C3,5 trimethoxyphenyl), 164.97 (C=O amide), 165.45 (C=O dimethoxybenzamide), 174.15 (C=O carboxylic). Anal. Calcd. for C_24_H_28_N_2_O_9_ (488.49): C, 59.01; H, 5.78; N, 5.73. Found: C, 59.09; H, 5.81; N, 5.66.

#### 3.2.4. General Procedure for the Preparation of (*Z*)-*N*-(3-Aryl-3-oxo-1-(3,4,5-Trimethoxyphenyl)prop-1-en-2-yl)-3,4-Dimethoxybenzamides **4a**–**c**

A mixture of 2-(3,4-dimethoxyphenyl)-4-(3,4,5-trimethoxybenzylidene)oxazolone **1** (399 mg, 1 mmol) and the appropriate aryl amine (1 mmol) was refluxed in glacial acetic acid (20 mL) in the presence of anhydrous sodium acetate (82 mg, 1 mmol) for 1–2 h. After refluxing time, the reaction mixture was cooled down, poured into crushed ice and filtered. The obtained solid was crystallized from DMF/H_2_O (1:1) to afford pure compound **4a**–**c**.

##### (*Z*)-3,4-Dimethoxy-*N*-(3-oxo-3-(*M*-tolylamino)-1-(3,4,5-Trimethoxyphenyl)prop-1-en-2-yl)benzamide (**4a**)

White powder (279 mg, 55.09%), m.p. 248–250 °C. ^1^H-NMR (400 MHz, DMSO-*d_6_*, δ ppm): 2.28 (s, 3*H*, CH_3_), 3.65 (s, 6*H*, 2OCH_3_), 3.66 (s, 3*H*, OCH_3_), 3.81 (s, 3*H*, OCH_3_), 3.83 (s, 3*H*, OCH_3_), 6.88 (d, *J* = 7.5 Hz, 1*H*, arom.CH), 7.02 (s, 2*H*, arom.CH), 7.08 (d, *J* = 8.5 Hz, 1*H*, arom.CH), 7.15 (s, 1*H*, olefinic CH), 7.19 (t, *J* = 7.7 Hz, 1*H*, arom.CH), 7.54 (d, *J* = 8.3 Hz, 2*H*, arom.CH), 7.69 (s, 1*H*, arom.CH), 7.72 (d, *J* = 8.6 Hz, 1*H*, arom.CH), 10.08 (s, 1*H*, NH), 10.20 (s, 1*H*, NH). ^13^C-NMR (100 MHz, DMSO-*d_6_*, δ ppm): 21.32 (CH_3_), 55.78 (OCH_3_), 55.81 (3OCH_3_), 60.26 (OCH_3_), 107.33 (C2,6 trimethoxyphenyl), 111.02 (C5 dimethoxybenzamide), 111.24 (C2 dimethoxybenzamide), 117.57 (C6 tolyl), 120.90 (C6 dimethoxybenzamide), 121.64 (C4 tolyl), 124.32 (C2 tolyl), 125.73 (C olefinic), 128.50 (C5 tolyl), 128.89 (C1 trimethoxyphenyl), 129.75 (C1 dimethoxybenzamide), 130.44 (C olefinic), 137.81 (C3 tolyl), 138.14 (C4 trimethoxyphenyl), 139.23 (C1 tolyl), 148.37 (C3 dimethoxybenzamide), 151.94 (C4 dimethoxybenzamide), 152.73 (C3,5 trimethoxyphenyl), 164.53 (C=O amide), 165.70 (C=O dimethoxybenzamide). Anal. Calcd. for C_28_H_30_N_2_O_7_ (506.55): C, 66.39; H, 5.97; N, 5.53. Found: C, 66.28; H, 6.05; N, 5.58.

##### (*Z*)-*N*-(3-(4-Hydroxyphenylamino)-3-oxo-1-(3,4,5-Trimethoxyphenyl)prop-1-en-2-yl)-3,4-Dimethoxybenzamide (**4b**)

White powder (253 mg, 49.77%), m.p. 248–250 °C. ^1^H-NMR (400 MHz, DMSO-*d_6_*, δ ppm): 3.64 (s, 6*H*, 2OCH_3_), 3.66 (s, 3*H*, OCH_3_), 3.80 (s, 3*H*, OCH_3_), 3.83 (s, 3*H*, OCH_3_), 6.71 (d, *J* = 8.8 Hz, 2*H*, arom.CH), 7.00 (s, 2*H*, arom.CH), 7.07 (d, *J* = 8.4 Hz, 1*H*, arom.CH), 7.16 (s, 1*H*, olefinic CH), 7.47 (d, *J* = 8.8 Hz, 2*H*, arom.CH), 7.65–7.75 (m, 2*H*, arom.CH), 9.93 (s, 1*H*, NH), 10.20 (s, 1*H*, NH). ^13^C-NMR (100 MHz, DMSO-*d_6_*, δ ppm): 55.63 (OCH_3_), 55.66 (2OCH_3_), 55.67 (OCH_3_), 60.09 (OCH_3_), 107.14 (C2,6 trimethoxyphenyl), 110.84 (C5 dimethoxybenzamide), 111.20 (C2 dimethoxybenzamide), 114.89 (C3,5 aminophenol), 121.49 (C6 dimethoxybenzamide), 122.01 (C2,6 aminophenol), 125.83 (C olefinic), 128.59 (C1 trimethoxyphenyl), 129.78 (C1 dimethoxybenzamide), 130.57 (C olefinic), 130.79 (C1 aminophenol), 137.87 (C4 trimethoxyphenyl), 148.19 (C3 dimethoxybenzamide), 151.71 (C4 dimethoxybenzamide), 152.57 (C3,5 trimethoxyphenyl), 153.63 (C4 aminophenol), 163.91 (C=O amide), 165.45 (C=O dimethoxybenzamide). Anal. Calcd. for C_27_H_28_N_2_O_8_ (508.52): C, 63.77; H, 5.55; N, 5.51. Found: C, 63.88; H, 5.45; N, 5.38.

##### (*Z*)-3,4-Dimethoxy-*N*-(3-(4-Methoxyphenylamino)-3-oxo-1-(3,4,5-Trimethoxyphenyl)prop-1-en-2-yl)benzamide (**4c**)

White powder (2.48 g, 58.61%), m.p. 248–250 °C. ^1^H-NMR (400 MHz, DMSO-*d_6_*, δ ppm): 3.64 (s, 6*H*, 2OCH_3_), 3.66 (s, 3*H*, OCH_3_), 3.73 (s, 3*H*, OCH_3_), 3.80 (s, 3*H*, OCH_3_), 3.83 (s, 3*H*, OCH_3_), 6.89 (d, *J* = 9.1 Hz, 2*H*, arom.CH), 7.01 (s, 2*H*, arom.CH), 7.07 (d, *J* = 8.5 Hz, 1*H*, arom.CH), 7.17 (s, 1*H*, olefinic CH), 7.63 (d, *J* = 9.0 Hz, 2*H*, arom.CH), 7.67–7.75 (m, 2*H*, arom.CH), 10.05 (s, 1*H*, NH), 10.19 (s, 1*H*, NH). ^13^C-NMR (100 MHz, DMSO-*d_6_*, δ ppm): 55.16 (OCH_3_), 55.62 (OCH_3_), 55.65 (3OCH_3_), 60.08 (OCH_3_), 107.15 (C2,6 trimethoxyphenyl), 110.84 (C5 dimethoxybenzamide), 111.17 (C2 dimethoxybenzamide), 113.60 (C3,5 methoxyphenyl), 121.48 (C6 dimethoxybenzamide), 121.75 (C2,6 methoxyphenyl), 125.76 (C olefinic), 128.63 (C1 trimethoxyphenyl), 129.72 (C1 dimethoxybenzamide), 130.48 (C olefinic), 132.42 (C1 methoxyphenyl), 137.90 (C4 trimethoxyphenyl), 148.19 (C3 dimethoxybenzamide), 151.72 (C4 dimethoxybenzamide), 152.56 (C3,5 trimethoxyphenyl), 155.31 (C4 methoxyphenyl), 164.09 (C=O amide), 165.44 (C=O dimethoxybenzamide). Anal. Calcd. for C_28_H_30_N_2_O_8_ (522.55): C, 64.36; H, 5.79; N, 5.36. Found: C, 64.44; H, 5.72; N, 5.27.

#### 3.2.5. General Procedure for the Preparation of (*Z*)-3,4-Dimethoxy-*N*-(3-oxo-3-(2-Phenylhydrazinyl)-1-(3,4,5-Trimethoxyphenyl)prop-1-en-2-yl)benzamide (**5**)

To a stirred suspension of 2-(3,4-dimethoxyphenyl)-4-(3,4,5-trimethoxybenzylidene)oxazolone **1** (399 mg, 1 mmol) in absolute ethanol, we added phenyl hydrazine (1 mmol), and the mixture was stirred at room temperature for 3 h. After evaporation under reduced pressure, the obtained crude product was crystallized from ethanol/H_2_O (3:1) to get pure compound **5**.

White powder (338 mg, 64.73%), m.p. 248–250 °C. ^1^H-NMR (400 MHz, DMSO-*d_6_*, δ ppm): 3.62 (s, 6*H*, 2OCH_3_), 3.65 (s, 3*H*, OCH_3_), 3.81 (s, 3*H*, OCH_3_), 3.83 (s, 3*H*, OCH_3_), 6.69 (t, *J* = 7.3 Hz, 1*H*, arom.CH), 6.84 (d, *J* = 7.8 Hz, 2*H*, arom.CH), 7.00 (s, 2*H*, arom.CH), 7.07 (d, *J* = 8.5 Hz, 1*H*, arom.CH), 7.14 (t, *J* = 7.8 Hz, 2*H*, arom.CH), 7.25 (s, 1*H*, olefinic CH), 7.67 (s, 1*H*, arom.CH), 7.71 (d, *J* = 8.5 Hz, 1*H*, arom.CH), 7.75 (d, *J* = 2.5 Hz, 1*H*, NH), 9.85 (s, 1*H*, NH), 10.03 (d, *J* = 2.5 Hz, 1*H*, NH). ^13^C-NMR (100 MHz, DMSO-*d_6_*, δ ppm): 56.08 (2OCH_3_), 56.13 (OCH_3_), 60.52 (OCH_3_), 107.67 (C2,6 trimethoxyphenyl), 111.23 (C5 dimethoxybenzamide), 111.74 (C2 dimethoxybenzamide), 112.92 (C2,6 phenyl), 118.79 (C4 phenyl), 121.97 (C6 dimethoxybenzamide), 126.37 (C olefinic), 129.00 (C3,5 phenyl), 129.18 (C1 trimethoxyphenyl), 129.93 (C1 dimethoxybenzamide), 129.97 (C olefinic), 138.45 (C4 trimethoxyphenyl), 148.57 (C3 dimethoxybenzamide), 149.98 (C1 phenyl), 152.14 (C4 dimethoxybenzamide), 153.02 (C3,5 trimethoxyphenyl), 165.69 (C=O hydrazide), 166.11 (C=O dimethoxybenzamide). Anal. Calcd. for C_27_H_29_N_3_O_7_ (507.54): C, 63.89; H, 5.76; N, 8.28. Found: C, 64.03; H, 5.87; N, 8.16.

#### 3.2.6. General Procedure for the Preparation of (*Z*)-*N*-(3-(2-Isonicotinoylhydrazinyl)-3-oxo-1-(3,4,5-Trimethoxyphenyl)prop-1-en-2-yl)-3,4-Dimethoxybenzamide (**6**)

To a stirred suspension of 2-(3,4-dimethoxyphenyl)-4-(3,4,5-trimethoxybenzylidene)oxazolone **1** (399 mg, 1 mmol) in absolute ethanol, we added isonicotinic acid hydrazide (137 mg, 1 mmol). The reaction mixture was refluxed for 5 h. The excess solvent was evaporated under vacuum to afford the crude product. This was further purified by crystallization from ethanol/H_2_O (3:1) to furnish pure compound **6**.

White powder (285 mg, 53.11%), m.p. 248–250 °C. ^1^H-NMR (400 MHz, DMSO-*d_6_*, δ ppm): 3.63 (s, 6*H*, 2OCH_3_), 3.66 (s, 3*H*, OCH_3_), 3.81 (s, 3*H*, OCH_3_), 3.83 (s, 3*H*, OCH_3_), 3.88 (s, 3*H*, OCH_3_), 7.00 (s, 2*H*, arom.CH), 7.07 (d, *J* = 8.5 Hz, 1*H*, arom.CH), 7.18–7.24 (m, 1*H*, arom.CH), 7.34 (s, 1*H*, olefinic CH), 7.71 (d, *J* = 6.0 Hz, 1*H*, arom.CH), 7.82 (d, *J* = 6.0 Hz, 2*H*, arom.CH), 8.77 (d, *J* = 5.7 Hz, 2*H*, arom.CH), 9.89 (s, 1*H*, NH), 10.31 (s, 1*H*, NH), 10.79 (s, 1*H*, NH). ^13^C-NMR (100 MHz, DMSO-*d_6_*, δ ppm): 55.63 (2OCH_3_), 55.66 (OCH_3_), 55.78 (OCH_3_), 60.07 (OCH_3_), 107.23 (C2,6 trimethoxyphenyl), 110.79 (C5 dimethoxybenzamide), 111.25 (C2 dimethoxybenzamide), 121.38 (C3,5 pyridyl), 121.52 (C6 dimethoxybenzamide), 125.81 (C olefinic), 128.10 (C1 trimethoxyphenyl), 129.28 (C1 dimethoxybenzamide), 130.74 (C olefinic), 138.17 (C4 trimethoxyphenyl), 139.68 (C4 pyridyl), 148.11 (C3 dimethoxybenzamide), 150.40 (C2,6 pyridyl), 151.70 (C4 dimethoxybenzamide), 152.60 (C3,5 trimethoxyphenyl), 163.99 (C=O isonicotinic acid hydrazide), 164.61 (C=O amide), 165.56 (C=O dimethoxybenzamide). Anal. Calcd. for C_27_H_28_N_4_O_8_ (536.53): C, 60.44; H, 5.26; N, 10.44. Found: C, 60.61; H, 5.36; N, 10.23.

#### 3.2.7. General Procedure for the Preparation of (*Z*)-1-Aryl-2-(3,4-Dimethoxyphenyl)-4-(3,4,5-Trimethoxybenzylidene)-1*H*-Imidazol-5(4*H*)-ones **7a**,**b**

A suspension of 2-(3,4-dimethoxyphenyl)-4-(3,4,5-trimethoxybenzylidene)oxazolone **1** (399 mg, 1 mmol) in glacial acetic acid (25 mL) containing anhydrous sodium acetate (98.4 mg, 1.2 mmol) was heated to reflux for 12 h. After refluxing time, the reaction mixture was cooled and poured into crushed ice. The obtained solid product was filtered off and crystallized from ethanol/H_2_O (3:1) to afford pure compound **7a**,**b**.

##### (*Z*)-2-(3,4-Dimethoxyphenyl)-1-(4-Hydroxyphenyl)-4-(3,4,5-Trimethoxybenzylidene)-1*H*-Imidazol-5(4*H*)-one (**7a**)

White powder (300 mg, 61.08%), m.p. 248–250 °C. ^1^H-NMR (400 MHz, DMSO-*d_6_*, δ ppm): 3.60 (s, 3*H*, OCH_3_), 3.75 (s, 3*H*, OCH_3_), 3.77 (s, 3*H*, OCH_3_), 3.87 (s, 6*H*, 2OCH_3_), 6.87 (d, *J* = 8.7 Hz, 2*H*, arom.CH), 6.96 (d, *J* = 8.7 Hz, 1*H*, arom.CH), 7.03 (dd, *J* = 8.5, 2.0 Hz, 1*H*, arom.CH), 7.11 (s, 1*H*, olefinic CH), 7.12 (d, *J* = 3.6 Hz, 2*H*, arom.CH), 7.31 (d, *J* = 1.9 Hz, 1*H*, arom.CH), 7.85 (s, 2*H*, arom.CH), 10.00 (s, 1*H*, OH). ^13^C-NMR (100 MHz, DMSO-*d_6_*, δ ppm): 54.92 (OCH_3_), 55.68 (OCH_3_), 55.75 (2OCH_3_), 60.24 (OCH_3_), 109.69 (C2,6 trimethoxybenzylidene), 111.20 (C5 dimethoxyphenyl), 111.57 (C2 dimethoxyphenyl), 116.05 (C3,5 hydroxyphenyl), 120.54 (C1 dimethoxyphenyl), 122.54 (C6 dimethoxyphenyl), 126.05 (C olefinic), 126.10 (C1 trimethoxybenzylidene), 129.42 (C2,6 hydroxyphenyl), 129.93 (C1 hydroxyphenyl), 137.70 (C4 imidazole), 139.49 (C4 trimethoxybenzylidene), 148.15 (C3 dimethoxyphenyl), 151.70 (C4 dimethoxyphenyl), 152.79 (C3,5 trimethoxybenzylidene), 157.77 (C4 hydroxyphenyl), 159.57 (C2 imidazole), 170.41 (C=O imidazole). Anal. Calcd. for C_27_H_26_N_2_O_7_ (490.50): C, 66.11; H, 5.34; N, 5.71. Found: C, 65.96; H, 5.42; N, 5.84.

##### (*Z*)-2-(3,4-Dimethoxyphenyl)-1-(4-Methoxyphenyl)-4-(3,4,5-Trimethoxybenzylidene)-1*H*-Imidazol-5(4*H*)-one (**7b**)

White powder (309 mg, 61.26%), m.p. 248–250 °C. ^1^H-NMR (400 MHz, DMSO-*d_6_*, δ ppm): 3.59 (s, 3*H*, OCH_3_), 3.75 (s, 3*H*, OCH_3_), 3.77 (s, 3*H*, OCH_3_), 3.81 (s, 3*H*, OCH_3_), 3.87 (s, 6*H*, 2OCH_3_), 6.96 (s, 1*H*, arom.CH), 7.01 (dd, *J* = 8.5, 1.8 Hz, 1*H*, arom.CH), 7.07 (d, *J* = 8.8 Hz, 2*H*, arom.CH), 7.13 (s, 1*H*, olefinic CH), 7.26 (d, *J* = 8.7 Hz, 2*H*, arom.CH), 7.30 (d, *J* = 1.7 Hz, 1*H*, arom.CH), 7.85 (s, 2*H*, arom.CH). ^13^C-NMR (100 MHz, DMSO-*d_6_*, δ ppm): 54.91 (OCH_3_), 55.48 (OCH_3_), 55.65 (OCH_3_), 55.72 (2OCH_3_), 60.21 (OCH_3_), 109.68 (C2,6 trimethoxybenzylidene), 111.18 (C2 dimethoxyphenyl), 111.53 (C5 dimethoxyphenyl), 114.70 (C3,5 methoxyphenyl), 120.47 (C1 dimethoxyphenyl), 122.47 (C6 dimethoxyphenyl), 126.15 (C olefinic), 127.60 (C1 trimethoxybenzylidene), 129.42 (C2,6 methoxyphenyl), 129.87 (C1 methoxyphenyl), 137.61 (C4 imidazole), 139.49 (C4 trimethoxybenzylidene), 148.14 (C3 dimethoxyphenyl), 151.66 (C4 dimethoxyphenyl), 152.75 (C3,5 trimethoxybenzylidene), 159.24 (C4 methoxyphenyl), 159.42 (C2 imidazole), 170.26 (C=O imidazole). Anal. Calcd. for C_28_H_28_N_2_O_7_ (504.53): C, 66.66; H, 5.59; N, 5.55. Found: C, 66.75; H, 5.51; N, 5.65.

#### 3.2.8. General Procedure for the Preparation of (*Z*)-3-(3,4-Dimethoxyphenyl)-6-oxo-5-(3,4,5-Trimethoxybenzylidene)-5,6-Dihydro-1,2,4-Triazine-2(1*H*)-Carbothioamide (**8**)

To a suspension of 2-(3,4-dimethoxyphenyl)-4-(3,4,5-trimethoxybenzylidene)oxazolone **1** (399 mg, 1 mmol) in glacial acetic acid (20 mL), we added thiosemicarbazide (91 mg, 1 mmol) and anhydrous sodium acetate (98.4 mg, 1.2 mmol). The resulting reaction mixture was heated to for 8 h. After refluxing time, a solid separated out that was filtered, dried and crystallized from ethanol/H_2_O (3:1) to furnish pure *N*-thioamide molecule **8**.

White powder (261.11 mg, 55.26%), m.p. 248–250 °C. ^1^H-NMR (400 MHz, DMSO-*d_6_*, δ ppm): 3.75 (s, 3*H*, OCH_3_), 3.83 (s, 3*H*, OCH_3_), 3.86 (s, 9H, 3OCH_3_), 7.09 (d, *J* = 19.1 Hz, 1*H*, arom.CH), 7.18 (d, *J* = 9.0 Hz, 1*H*, arom.CH), 7.60–7.75 (m, 2*H*, arom.CH and olefinic CH), 7.82 (s, 2*H*, arom.CH), 8.34 (d, *J* = 50.8 Hz, 2*H*, 2NH), 10.25 (s, 1*H*, NH). ^13^C-NMR (100 MHz, DMSO-*d_6_*, δ ppm): 55.64 (OCH_3_), 55.68 (2OCH_3_), 55.71 (OCH_3_), 60.08 (OCH_3_), 107.41 (C2,6 trimethoxybenzylidene), 110.94 (C2 dimethoxyphenyl), 111.20 (C5 dimethoxyphenyl), 121.73 (C1 dimethoxyphenyl), 124.97 (C6 dimethoxyphenyl), 127.97 (C olefinic), 129.05 (C1 trimethoxybenzylidene), 134.29 (C5 triazinone), 138.18 (C4 trimethoxybenzylidene), 148.18 (C3 dimethoxyphenyl), 152.12 (C4 dimethoxyphenyl), 152.62 (C3,5 trimethoxybenzylidene), 164.53 (C3 triazinone), 166.68 (C=O triazinone), 181.05 (C=S). Anal. Calcd. for C_22_H_24_N_4_O_6_S (472.51): C, 55.92; H, 5.12; N, 11.86. Found: C, 56.04; H, 5.03; N, 12.03.

#### 3.2.9. General Procedure for the Preparation of (*Z*)-3-(3,4-Dimethoxyphenyl)-2-Phenyl-5-(3,4,5-Trimethoxybenzylidene)-1,2-Dihydro-1,2,4-Triazin-6(5*H*)-one (**9**)

To a suspension of 2-(3,4-dimethoxyphenyl)-4-(3,4,5-trimethoxybenzylidene)oxazolone **1** (399 mg, 1 mmol) in pure ethanol (20 mL), we added phenyl hydrazine (1 mmol), and the reaction mixture was refluxed for 6 h. After refluxing time, the solvent was evaporated under pressure and the crude solid thus obtained was filtered, washed with water, dried and crystallized from ethanol/H_2_O (3:1) to afford pure compound **9**.

White powder (340 mg, 69.39%), m.p. 248–250 °C. ^1^H-NMR (400 MHz, DMSO-*d_6_*, δ ppm): 3.71 (s, 3*H*, OCH_3_), 3.77 (s, 3*H*, OCH_3_), 3.81 (s, 3*H*, OCH_3_), 3.88 (s, 6*H*, 2OCH_3_), 6.71 (d, *J* = 7.9 Hz, 2*H*, arom.CH), 6.83 (t, *J* = 7.3 Hz, 1*H*, arom.CH), 7.08 (d, *J* = 8.7 Hz, 1*H*, arom.CH), 7.15 (s, 1*H*, olefinic CH), 7.22 (t, *J* = 7.9 Hz, 2*H*, arom.CH), 7.85 (d, *J* = 1.9 Hz, 1*H*, arom.CH), 7.88 (s, 2*H*, arom.CH), 7.94 (dd, *J* = 8.6, 1.9 Hz, 1*H*, arom.CH), 9.07 (s, 1*H*, NH). ^13^C-NMR (100 MHz, DMSO-*d_6_*, δ ppm): 55.45 (OCH_3_), 56.16 (3OCH_3_), 60.69 (OCH_3_), 110.43 (C2,6 trimethoxybenzylidene), 111.40 (C2 dimethoxyphenyl), 111.94 (C5 dimethoxyphenyl), 112.66 (C2,6 phenyl), 120.13 (C1 dimethoxyphenyl), 120.51 (C4 phenyl), 122.75 (C6 dimethoxyphenyl), 127.17 (C olefinic), 129.82 (C3,5 phenyl), 130.14 (C1 trimethoxybenzylidene), 135.94 (C5 triazinone), 140.16 (C4 trimethoxybenzylidene), 146.98 (C1 phenyl), 148.94 (C3 dimethoxyphenyl), 152.90 (C4 dimethoxyphenyl), 153.23 (C3,5 trimethoxybenzylidene), 159.50 (C3 triazinone), 169.77 (C=O triazinone). Anal. Calcd. for C_27_H_27_N_3_O_6_ (489.52): C, 66.25; H, 5.56; N, 8.58. Found: C, 66.32; H, 5.60; N, 8.51.

#### 3.2.10. General Procedure for the Preparation of (*Z*)-3-(3,4-Dimethoxyphenyl)-2-Isonicotinoyl-5-(3,4,5-Trimethoxybenzylidene)-1,2-Dihydro-1,2,4-Triazin-6(5*H*)-one (**10**)

A mixture of 2-(3,4-dimethoxyphenyl)-4-(3,4,5-trimethoxybenzylidene)oxazolone **1** (399 mg, 1 mmol), isonicotinic acid hydrazide (137 mg, 1 mmol) and sodium acetate anhydrous (98.4 mg, 1.2 mmol) in glacial acetic acid (20 mL) was heated to reflux for 12 h. After refluxing time, the solvent was concentrated under vacuum and then water was added to the residue. The crude solid thus obtained was filtered, washed with water, dried and crystallized from DMF/H_2_O (1:1) to furnish pure compound **10**.

White powder (308 mg, 59.42%), m.p. 248–250 °C. ^1^H-NMR (400 MHz, DMSO-*d_6_*, δ ppm): 3.71 (s, 3*H*, OCH_3_), 3.75 (s, 3*H*, OCH_3_), 3.79 (s, 3*H*, OCH_3_), 3.87 (s, 6*H*, 2OCH_3_), 7.05 (s, 1*H*, arom.CH), 7.07 (s, 1*H*, olefinic CH), 7.84 (s, 2*H*, arom.CH), 7.87 (d, *J* = 1.7 Hz, 2*H*, arom.CH), 7.89 (d, *J* = 7.5 Hz, 2*H*, arom.CH), 8.70 (d, *J* = 5.9 Hz, 2*H*, arom.CH), 10.03 (s, 1*H*, NH). ^13^C-NMR (100 MHz, DMSO-*d_6_*, δ ppm): 55.12 (OCH_3_), 55.65 (OCH_3_), 55.71 (2OCH_3_), 60.22 (OCH_3_), 109.68 (C2,6 trimethoxybenzylidene), 110.91 (C2 dimethoxyphenyl), 111.36 (C5 dimethoxyphenyl), 121.33 (C1 dimethoxyphenyl), 121.65 (C3,5 pyridyl), 121.95 (C6 dimethoxyphenyl), 125.30 (C olefinic), 130.05 (C1 trimethoxybenzylidene), 136.79 (C5 triazinone), 139.30 (C4 trimethoxybenzylidene), 148.34 (C3 dimethoxyphenyl), 150.09 (C2,6 pyridyl), 150.39 (C4 pyridyl), 152.00 (C4 dimethoxyphenyl), 152.74 (C3,5 trimethoxybenzylidene), 160.08 (C3 triazinone), 168.52 (C=O triazinone), 172.36 (C=O isonicotinamide). Anal. Calcd. for C_27_H_26_N_4_O_7_ (518.52): C, 62.54; H, 5.05; N, 10.81. Found: C, 62.44; H, 4.99; N, 10.94.

#### 3.2.11. General Procedure for the Preparation of (*Z*)-3-(3,4-Dimethoxyphenyl)-2-(4-Phenylthiazol-2-yl)-5-(3,4,5-Trimethoxybenzylidene)-1,2-Dihydro-1,2,4-Triazin-6(5*H*)-one (**11**)

A suspension of 2-(3,4-dimethoxyphenyl)-4-(3,4,5-trimethoxybenzylidene)oxazolone **1** (399 mg, 1 mmol) in DMF (20 mL) containing glacial acetic acid (0.5 mL) was added to 2-hydrazinyl-4-phenylthiazole (191 mg, 1 mmol). The reaction mixture was refluxed for 24 h. After cooling, the solution was poured into 100 mL of diethyl ether. The organic layer was washed with water (70 mL) and dried with Na_2_SO_4_. The solvent was evaporated under pressure and the crude product was crystallized from DMF/H_2_O (1:1) to get pure compound **11**.

White powder (293 mg, 51.25%), m.p. 248–250 °C. ^1^H-NMR (400 MHz, DMSO-*d_6_*, δ ppm): 3.76 (s, 6*H*, 2OCH_3_), 3.82 (s, 3*H*, OCH_3_), 3.88 (s, 6*H*, 2OCH_3_), 7.14 (d, *J* = 8.6 Hz, 1*H*, arom.CH), 7.21 (s, 1*H*, olefinic CH), 7.28 (t, *J* = 7.1 Hz, 1*H*, arom.CH), 7.36 (d, *J* = 7.6 Hz, 2*H*, arom.CH), 7.39 (s, 1*H*, thiazole CH), 7.76 (d, *J* = 7.7 Hz, 2*H*, arom.CH), 7.79–7.83 (m, 1*H*, arom.CH), 7.86 (d, *J* = 1.9 Hz, 1*H*, arom.CH), 7.88 (s, 2*H*, arom.CH), 10.80 (s, 1*H*, NH). ^13^C-NMR (100 MHz, DMSO-*d_6_*, δ ppm): 55.13 (OCH_3_), 55.72 (3OCH_3_), 60.24 (OCH_3_), 104.94 (C5 thiazole), 110.09 (C2,6 trimethoxybenzylidene), 110.74 (C2 dimethoxyphenyl), 111.61 (C5 dimethoxyphenyl), 119.39 (C1 dimethoxyphenyl), 121.93 (C6 dimethoxyphenyl), 125.61 (C2,6 phenyl), 127.46 (C4 phenyl), 127.84 (C olefinic), 128.64 (C3,5 phenyl), 129.44 (C1 trimethoxybenzylidene), 134.05 (C1 phenyl), 134.87 (C5 triazinone), 139.88 (C4 trimethoxybenzylidene), 148.62 (C3 dimethoxyphenyl), 150.57 (C4 thiazole), 152.51 (C4 dimethoxyphenyl), 152.78 (C3,5 trimethoxybenzylidene), 157.96 (C3 triazinone), 167.94 (C=O triazinone), 168.49 (C2 thiazole). Anal. Calcd. for C_30_H_28_N_4_O_6_S (572.63): C, 62.92; H, 4.93; N, 9.78. Found: C, 63.05; H, 5.02; N, 9.68.

### 3.3. Biological Evaluation

#### 3.3.1. MTT Cytotoxicity Assay

MTT colorimetric assay was carried out to investigate the impact of the newly synthesized compounds **2**–**11** on hepatocellular carcinoma (HepG2) and normal liver cell line (HL-7702) cell lines. See Section 3.3.1 in Supplementary Material.

#### 3.3.2. Tubulin Polymerization Assay

To explore the cytotoxic activity of the prepared compounds, tubulin polymerization assay of the most potent compounds **9**, **10** and **11** was performed according to the reported method [2]. See Section 3.3.2 in Supplementary Material.

#### 3.3.3. Cell Cycle Analysis

Cell cycle analysis in HepG2 cells was carried out by FACS analysis according to the manufacturer’s directions. See Section 3.3.3 in Supplementary Material.

#### 3.3.4. Annexin V/FITC Staining Assay

Annexin V/FITC double staining analysis in HepG2 cells was carried out by FACS analysis according to the manufacturer’s directions. See Section 3.3.4 in Supplementary Material.

#### 3.3.5. Mitochondrial Membrane Potential

MMP was measured by flow cytometry analysis in HepG2 cells according to the manufacturer’s directions. See Section 3.3.5 in Supplementary Material.

#### 3.3.6. Effect on p53, Bax and Bcl-2

Apoptotic markers (p53, Bax and Bcl-2) were measured in HepG2 cells using ELISA analysis according to the manufacturer’s directions. See Section 3.3.6 in Supplementary Material.

#### 3.3.7. Molecular Docking Study

All the docking study was performed using molecular operating environment (MOE 2009.10). See Section 3.3.7 in Supplementary Material.

## 4. Conclusions

In conclusion, a series of novel trimethoxyphenyl-based analogs were synthesized by varying the azalactone ring of 2-(3,4-dimethoxyphenyl)-4-(3,4,5-trimethoxybenzylidene)oxazolone **1**, and they were tested for their cytotoxic activity against the hepatocellular carcinoma HepG2 cell line and the normal liver HL-7702 cell line. The tested molecules exhibited good activity over the HepG2 cells, especially compounds **9**, **10**, and **11** with IC_50_ values of 1.38 ± 0.15, 2.52 ± 0.24, and 3.21 ± 0.22 μM, respectively, compared to podo (IC_50_ = 2.08 ± 0.04 μM). In addition, compounds **9**, **10**, and **11** exhibited β-tubulin polymerization inhibition percentage well, with percentage inhibitions of 86.73%, 80.51%, and 69.51%. Additionally, cell cycle analysis of compound **9** revealed cell cycle disturbance at the G2/M phase (3-fold more than control HepG2). Furthermore, the Annexin V-FITC/PI dual staining assay results show that treatment of HepG2 cells with compound **9** inducted a significant increase in Annexin-V-positive cells by almost 18-fold compared with the untreated control. Moreover, Compound **9** was further studied regarding its apoptotic potential in HepG2 cells; it decreased the level of MMP and Bcl-2 by 3.12- and 5.58-fold, respectively, compared with control HepG2 cells. It also boosted the level of p53 and Bax by 8.91- and 4.50-fold, respectively, compared with control HepG2 cells.

## Figures and Tables

**Table 1:** Cytotoxic screening of the tested trimethoxyphenyl-based analogues **2a**–**11**. Values are are the mean ± SE.

Comp No	IC_50_ Value (μM)	
	HepG2	HL-7702
**2a**	14.39 ± 0.82	NT
**2b**	11.09 ± 0.77	NT
**3a**	8.13 ± 0.54	NT
**3b**	19.41 ± 1.43	NT
**4a**	45.53 ± 2.89	NT
**4b**	17.69 ± 1.10	NT
**4c**	23.72 ± 1.37	NT
**5**	9.24 ± 0.52	NT
**6**	8.74 ± 0.57	NT
**7a**	4.53 ± 0.29	NT
**7b**	6.45 ± 0.24	NT
**8**	4.02 ± 0.09	NT
**9**	1.38 ± 0.15	29.07 ± 2.03
**10**	2.52 ± 0.24	NT
**11**	3.21 ± 0.22	NT
podo	2.08 ± 0.04	18.59 ± 0.58

NT: not tested.

## Data Availability

All data are available within the manuscript.

## Supplementary Materials

The following supporting information can be downloaded at: https://www.mdpi.com/article/10.3390/molecules27144621/s1, Figure S1: ^1^H-NMR spectrum of compound **1**, Figure S2: ^13^C-NMR spectrum of compound **1**, Figure S3: ^1^H-NMR spectrum of compound **2a**, Figure S4: ^13^C-NMR spectrum of compound **2a**, Figure S5: ^1^H-NMR spectrum of compound **2b**, Figure S6: ^13^C-NMR spectrum of compound **2b**, Figure S7: ^1^H-NMR spectrum of compound **3a**, Figure S8: ^13^C-NMR spectrum of compound **3a**, Figure S9: ^1^H-NMR spectrum of compound **3a**, Figure S10: ^13^C-NMR spectrum of compound **3b**, Figure S11: ^1^H-NMR spectrum of compound **4a**, Figure S12: ^13^C-NMR spectrum of compound **4a**, Figure S13: ^1^H-NMR spectrum of compound **4b**, Figure S14: ^13^C-NMR spectrum of compound **4b**, Figure S15: ^1^H-NMR spectrum of compound **4c**, Figure S16: ^13^C-NMR spectrum of compound **4c**, Figure S17: ^1^H-NMR spectrum of compound **5**, Figure S18: ^13^C-NMR spectrum of compound **5**, Figure S19: ^1^H-NMR spectrum of compound **6**, Figure S20: ^13^C-NMR spectrum of compound **6**, Figure S21: ^1^H-NMR spectrum of compound **7a**, Figure S22: ^13^C-NMR spectrum of compound **7a**, Figure S23: ^1^H-NMR spectrum of compound **7b**, Figure S24: ^13^C-NMR spectrum of compound **7b**, Figure S25: ^1^H-NMR spectrum of compound **8**, Figure S26: ^13^C-NMR spectrum of compound **8**, Figure S27: ^1^H-NMR spectrum of compound **9**, Figure S28: ^13^C-NMR spectrum of compound **9**, Figure S29: ^1^H-NMR spectrum of compound **10**, Figure S30: ^13^C-NMR spectrum of compound **10**, Figure S31: ^1^H-NMR spectrum of compound **11**, Figure S32: ^13^C-NMR spectrum of compound **11**.
