# Supplementary material for: Synthesis and Biological Activity Screening of Newly Synthesized Trimethoxyphenyl-Based Analogues as Potential Anticancer Agents
Source: Molecules. 2022 Jul 20;27(14):4621. doi: 10.3390/molecules27144621 (PMC9322052; doi:10.3390/molecules27144621)
Supplement: Supplementary file 1 [file molecules-27-04621-s001.zip › molecules-1810642-supplementary.pdf]

Supporting Information

Synthesis and Biological Activity Screening of Newly Synthesized Trimethoxyphenyl-Based Analogues as Potential Anticancer Agents

Tarfah Al-Warhi [1], Matokah Abualnaja [2], Ola A. Abu Ali [3], Fayez Althobaiti [4], Fahad Alharthi [5], Fahmy G. Elsaid [6,7], Ali A. Shati [6], Eman Fayad [4], Doaa Elghareeb [8,9], Ali H. Abu Almaaty [10] and Islam Zaki [11,*]

[1] Department of Chemistry, College of Science, Princess Nourah bint Abdulrahman University, P.O. Box 84428, Riyadh 11671, Saudi Arabia; tarfah-w@hotmail.com
[2] Department of Chemistry, Faculty of Applied Science, Umm Al-Qura University, Makkah Al Mukarrama 24381, Saudi Arabia; mmabualnaja@uqu.edu.sa
[3] Department of Chemistry, College of Science, Taif University, P.O. Box 11099, Taif 21944, Saudi Arabia; o.abuali@tu.edu.sa
[4] Department of Biotechnology, Faculty of Sciences, Taif University, P.O. Box 11099, Taif 21944, Saudi Arabia; faiz@tu.edu.sa (F.A.); e.esmail@tu.edu.sa (E.F.)
[5] Department of Biology, College of Science, Taif University, Saudi Arabia; f.alharthi@tu.edu.sa
[6] Biology Department, Science College, King Khalid University Abha 61421, Saudi Arabia; felsaid@kku.edu.sa (F.G.E.); aaalshati@kku.edu.sa (A.A.S.)
[7] Zoology Department, Faculty of Science, Mansoura University, Mansoura, 35516, Egypt
[8] Department of Biology, Jumum College University, Umm Al-Qura University, P.O Box 7388, Makkah 21955, Saudi Arabia; dekeshek@uqu.edu.sa
[9] Agriculture Genetic Engineering Research Institute (AGERI), Agriculture Research Centre, Egypt
[10] Zoology Department, Faculty of Science, Port Said University, Port Said 42526, Egypt; ali_zoology_2010@yahoo.com
[11] Pharmaceutical Organic Chemistry Department, Faculty of Pharmacy, Port Said University, Port Said 42526, Egypt
* Correspondence: eslam.zaki@pharm.psu.edu.eg

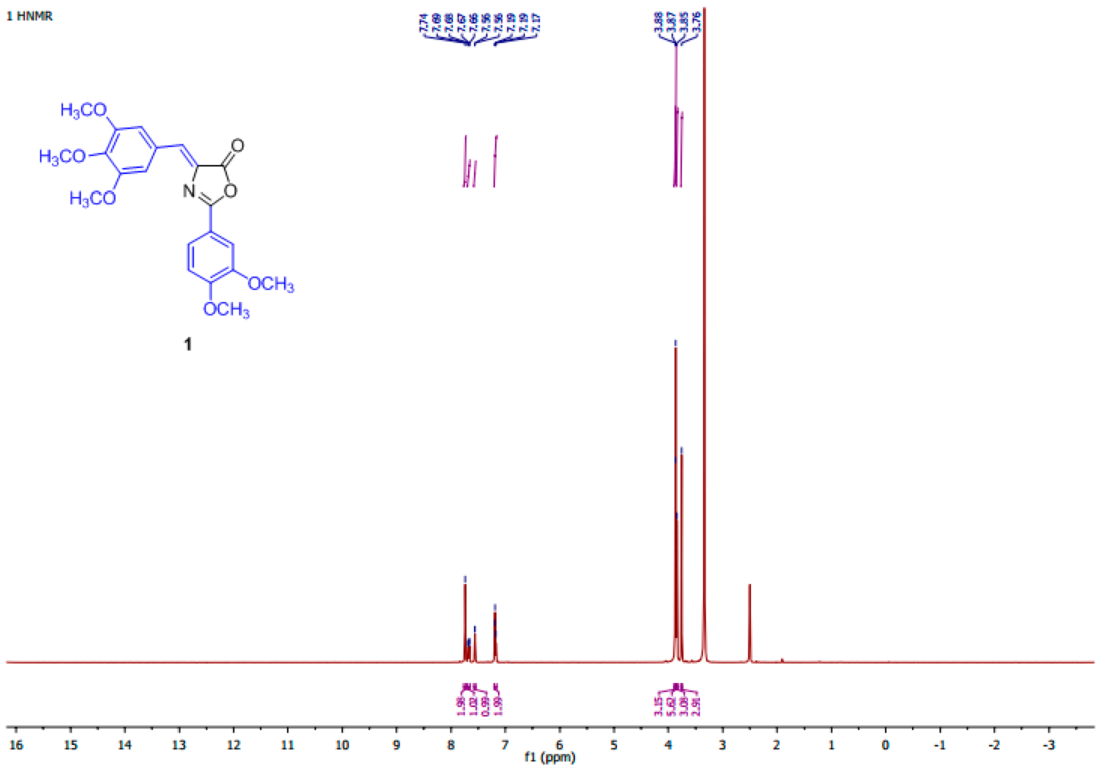

Figure S1. ^{1}H-NMR spectrum of compound **1**.

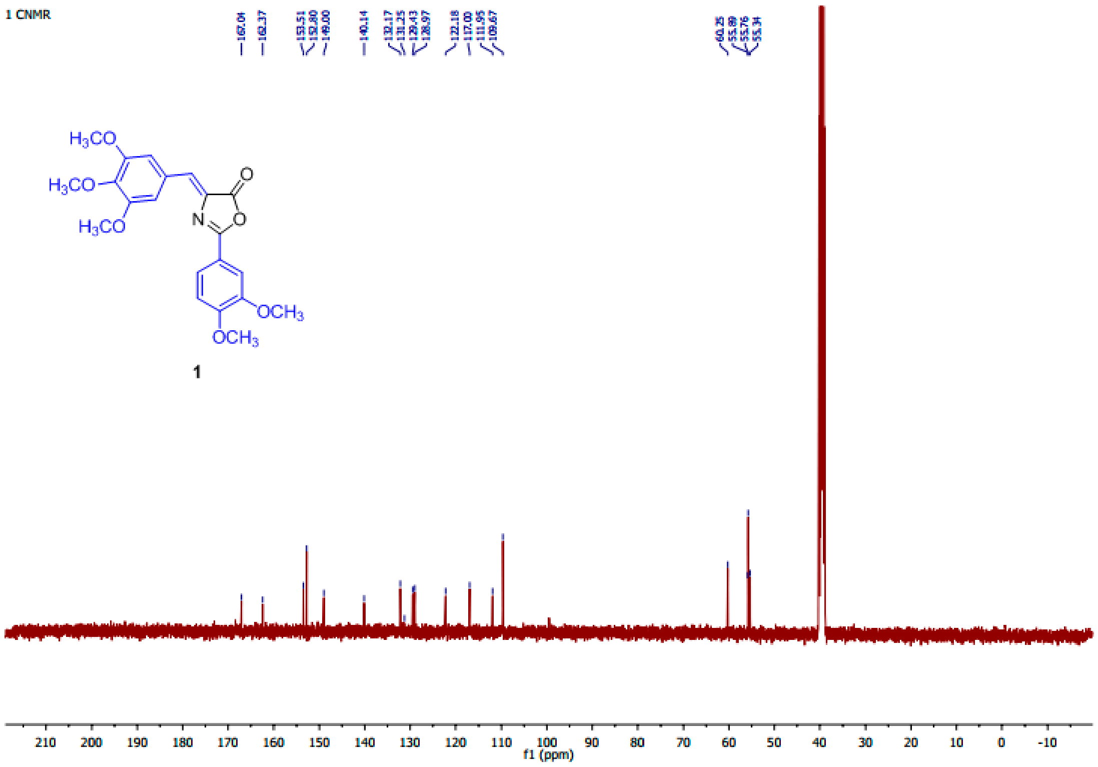

Figure S2. ^{13}C-NMR spectrum of compound **1**.

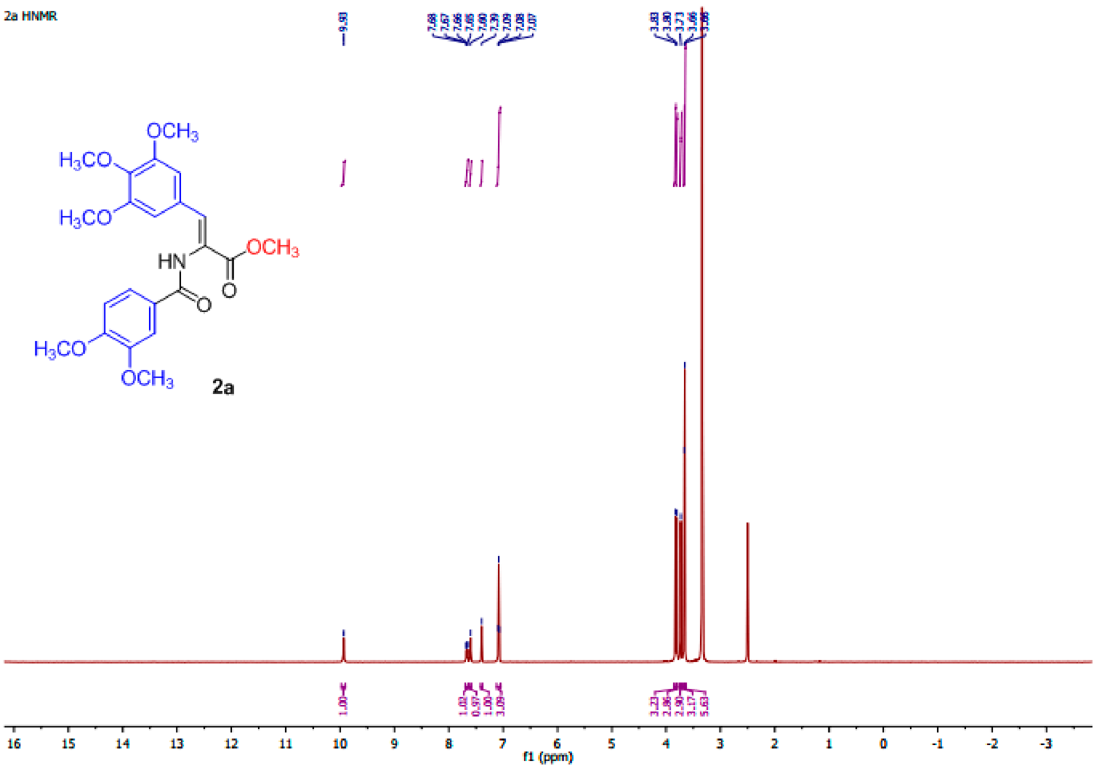

Figure S3. ^{1}H-NMR spectrum of compound **2a**.

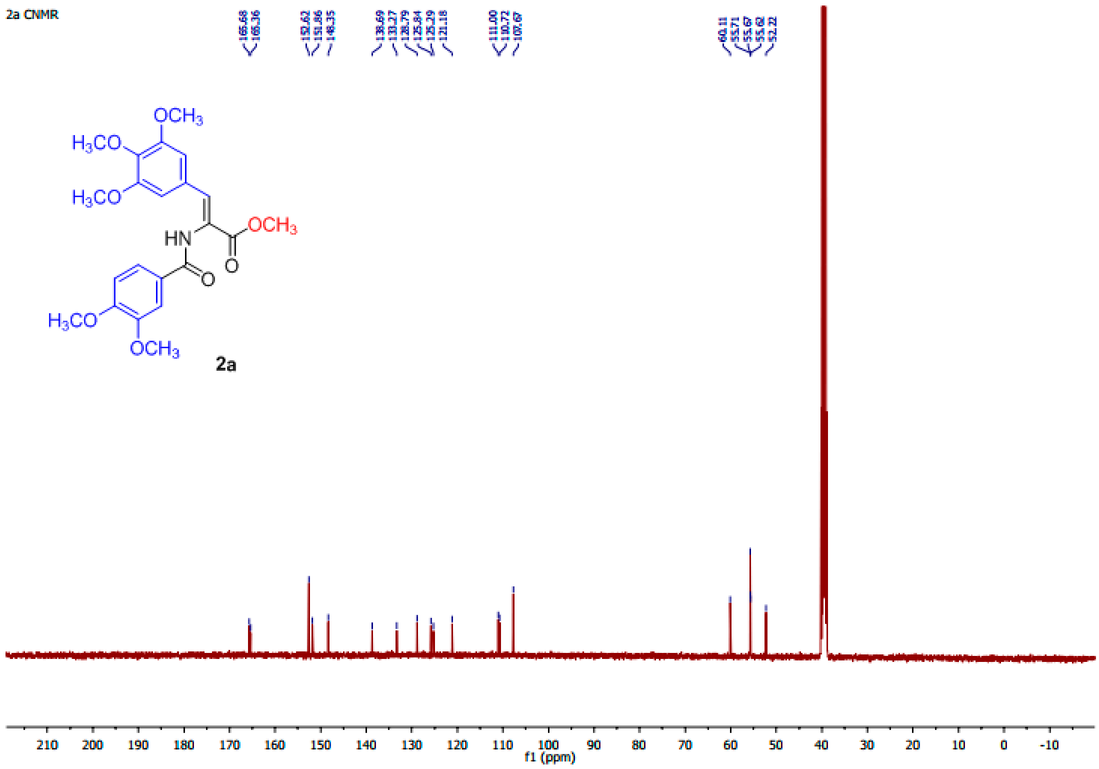

Figure S4. ^{13}C-NMR spectrum of compound **2a**.

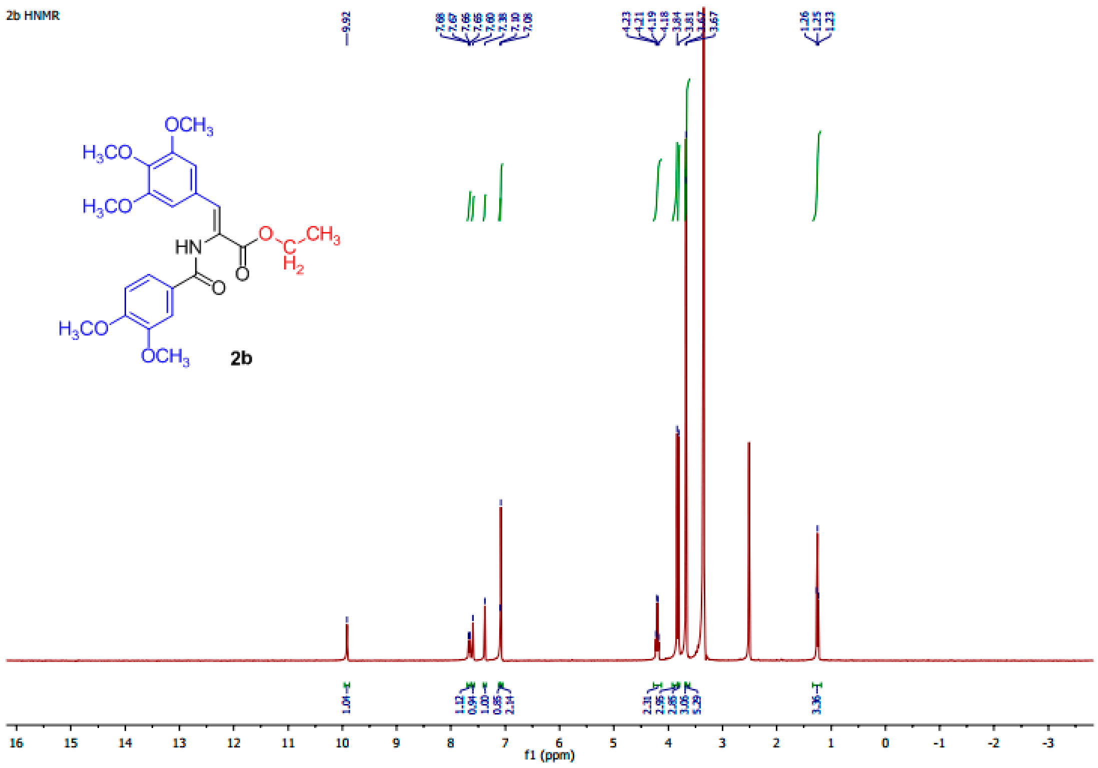

Figure S5. ^{1}H-NMR spectrum of compound **2b**.

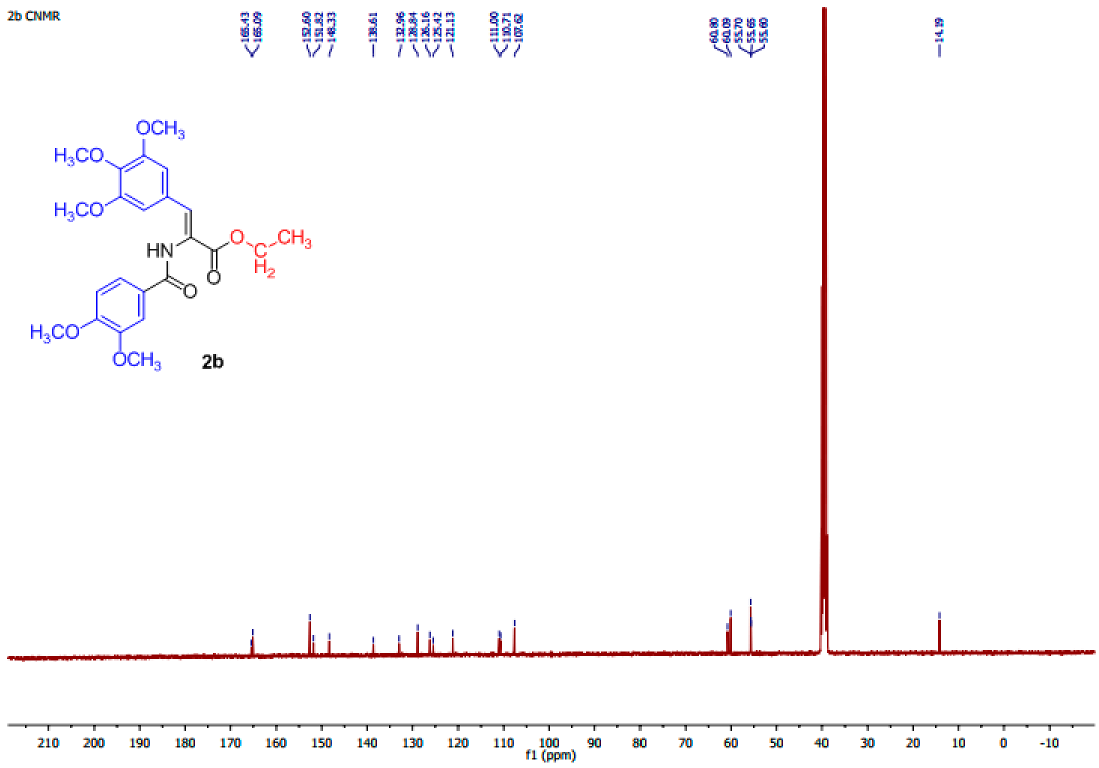

Figure S6. ^{13}C-NMR spectrum of compound **2b**.

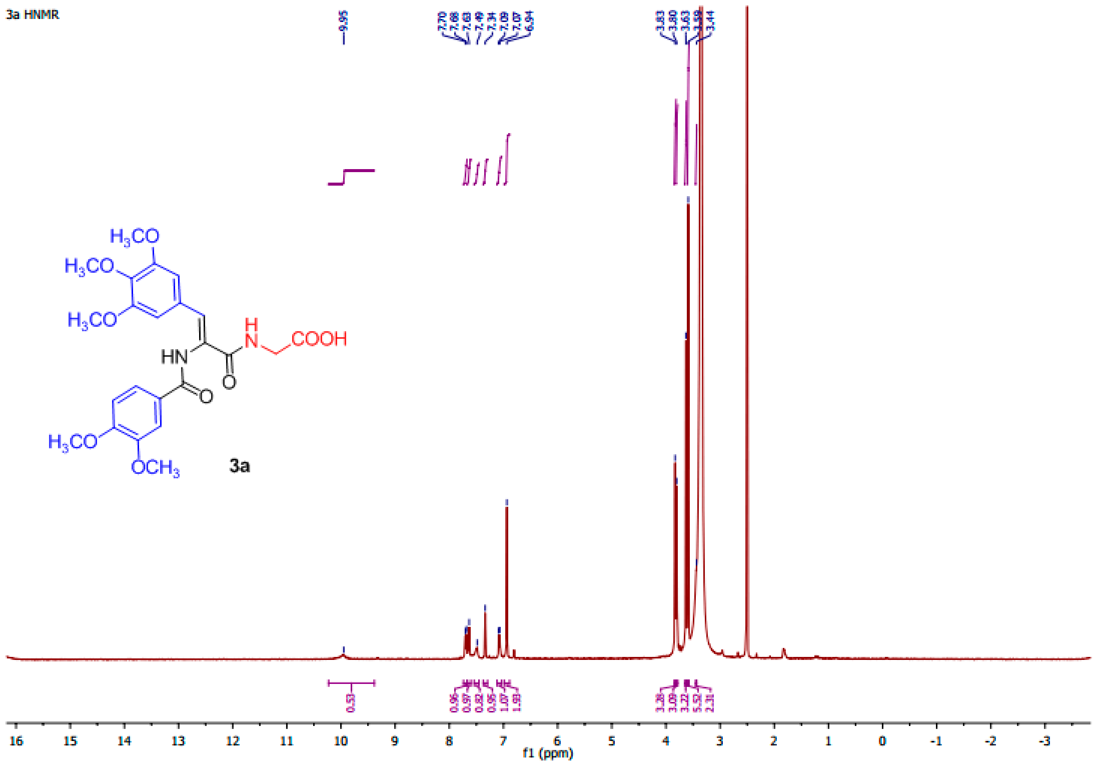

Figure S7. ^{1}H-NMR spectrum of compound **3a**.

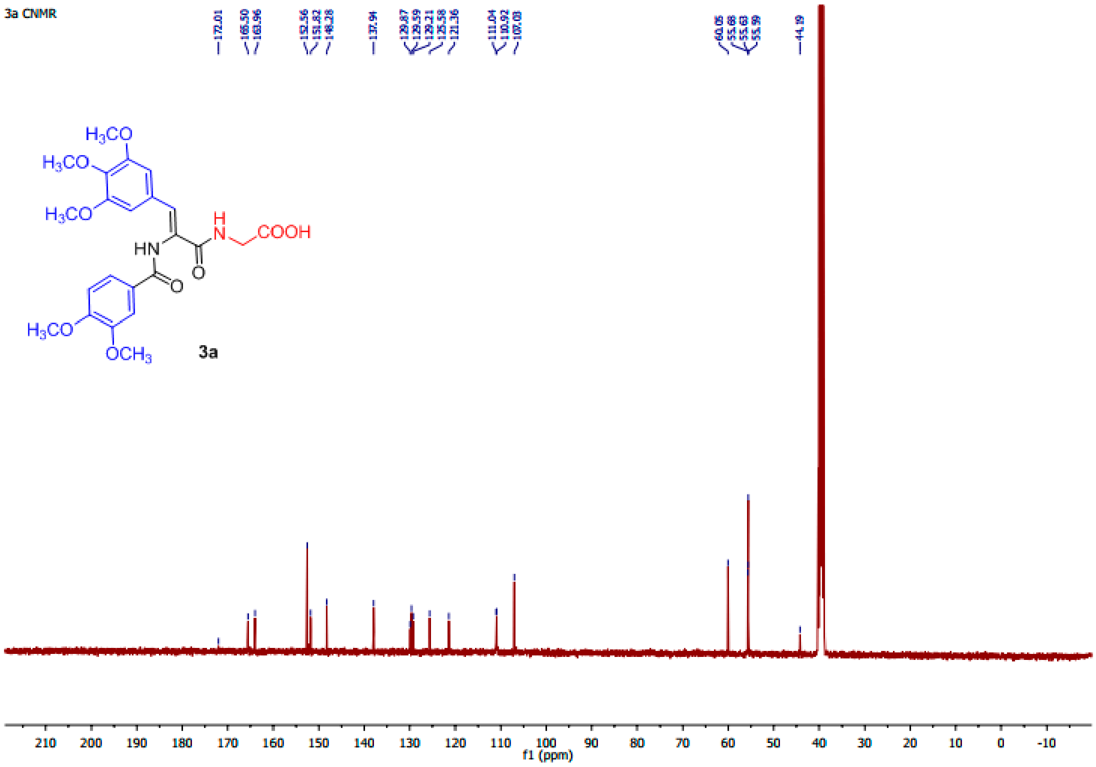

Figure S8. ^{13}C-NMR spectrum of compound **3a**.

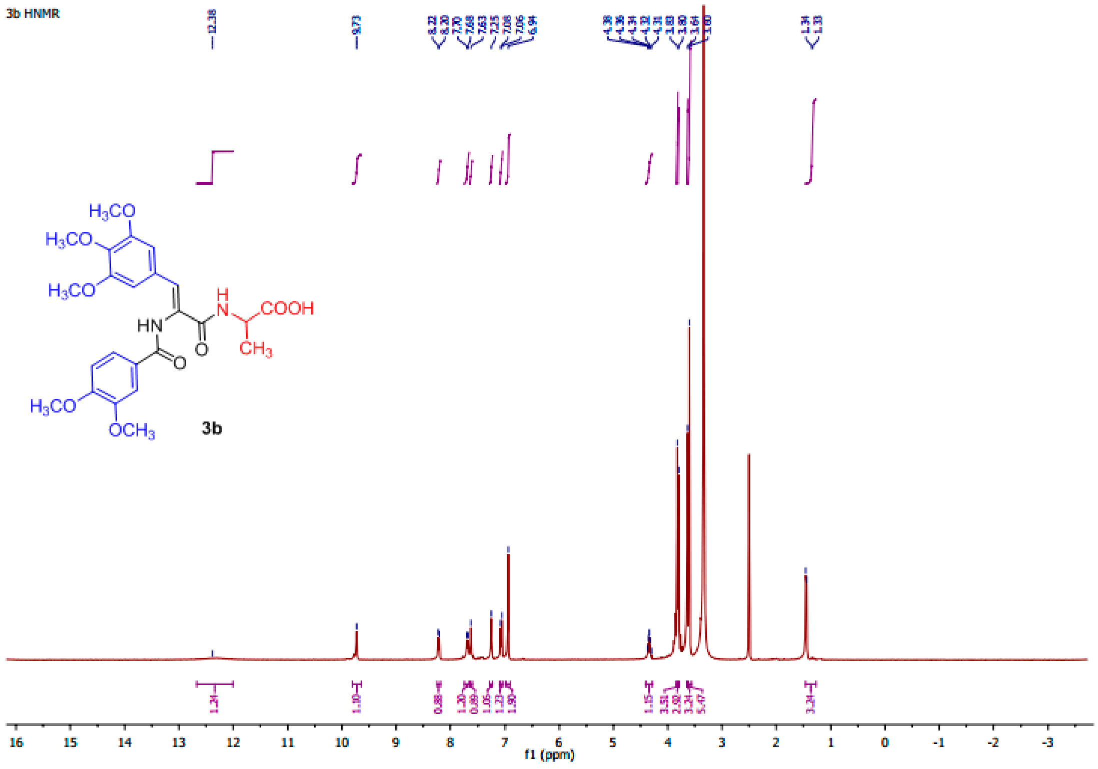

Figure S9. ^{1}H-NMR spectrum of compound **3b**.

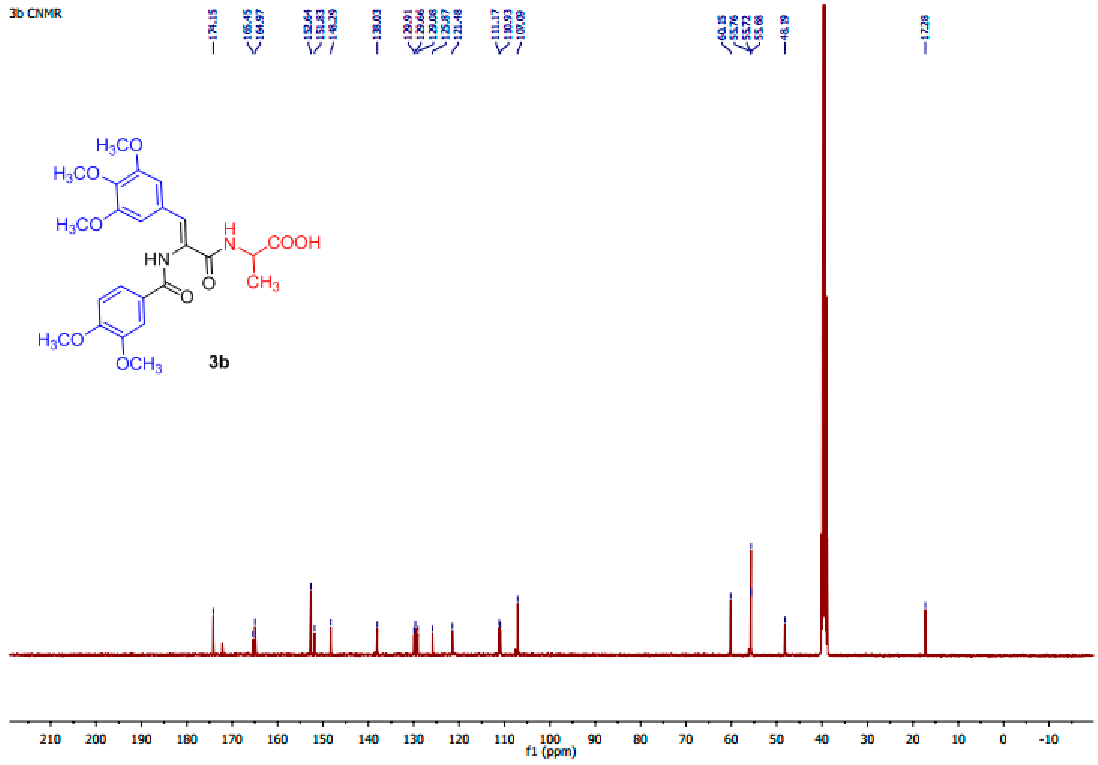

Figure S10. ^{13}C-NMR spectrum of compound **3b**.

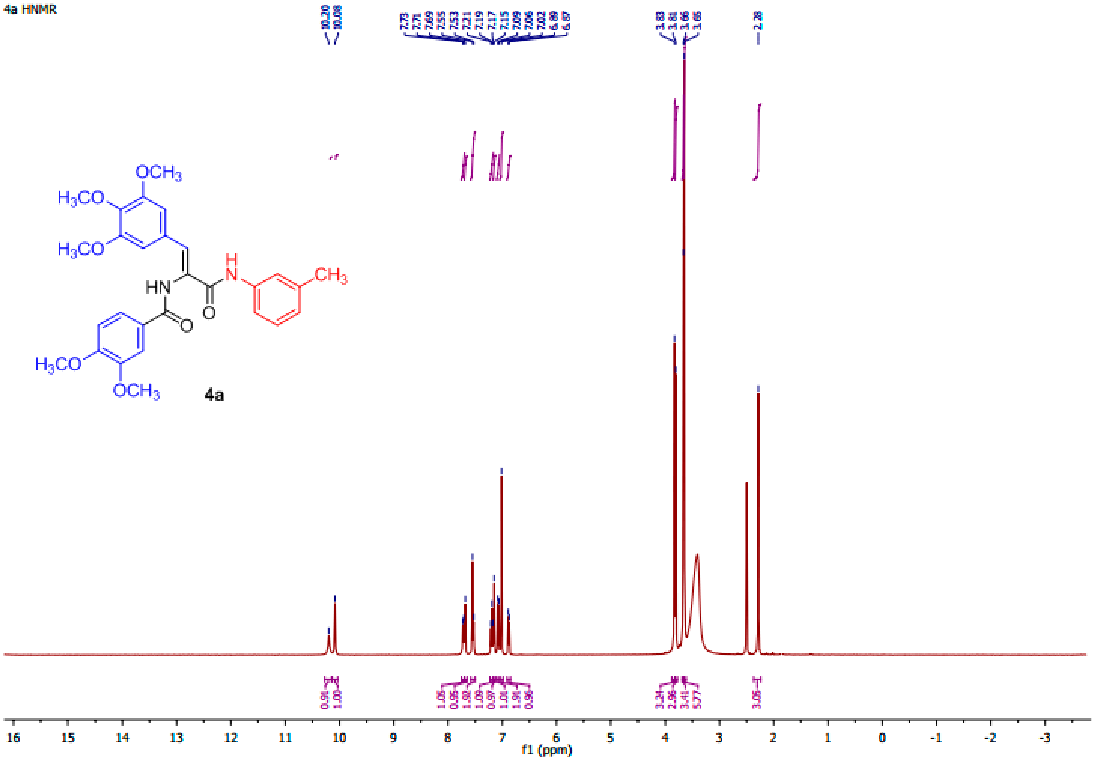

Figure S11. ^{1}H-NMR spectrum of compound **4a**.

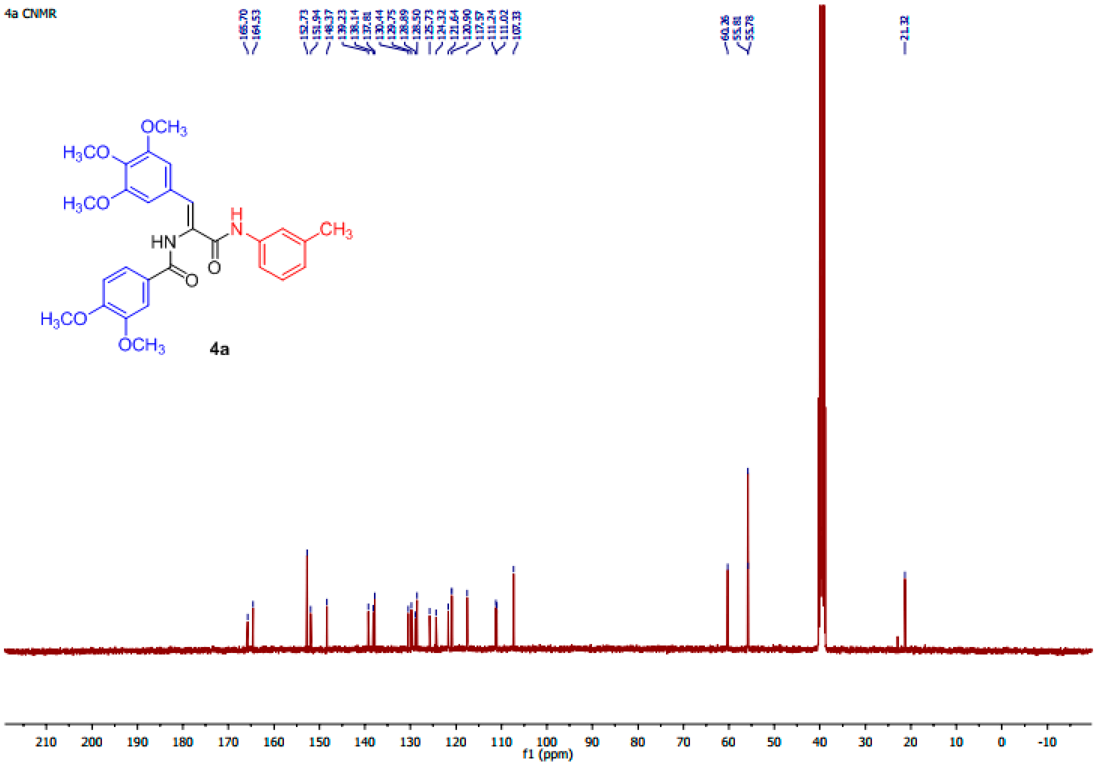

Figure S12. ^{13}C-NMR spectrum of compound **4a**.

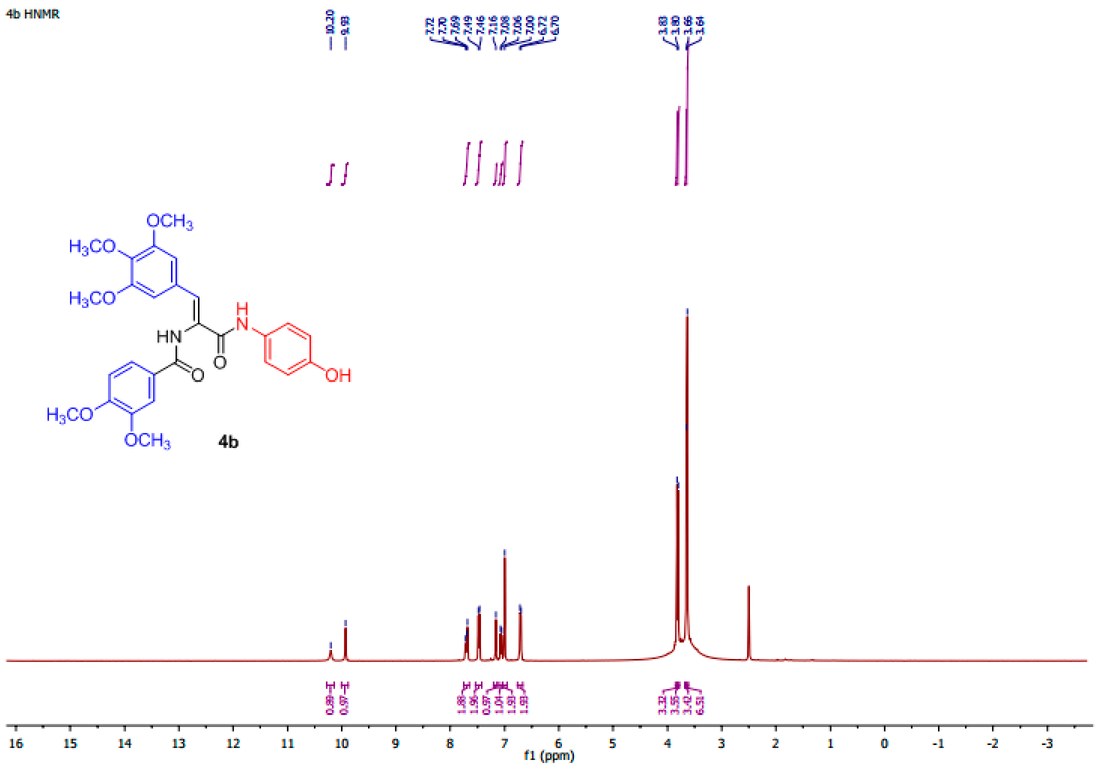

Figure S13. ^{1}H-NMR spectrum of compound **4b**.

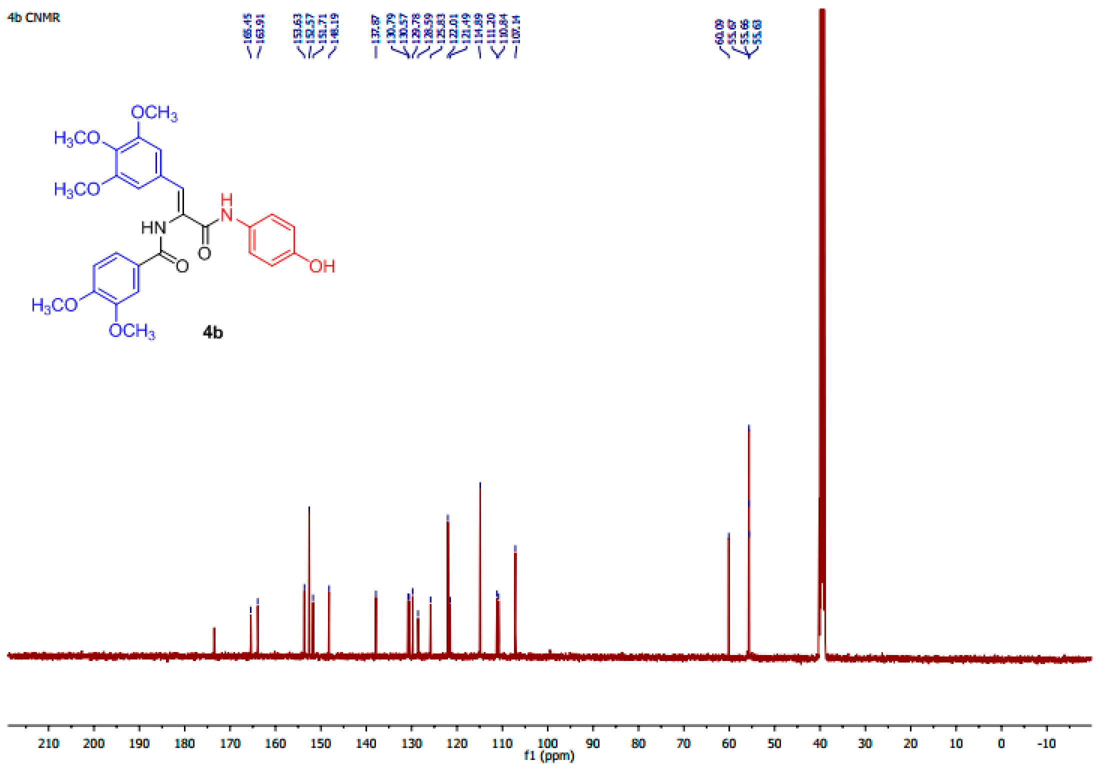

Figure S14. ^{13}C-NMR spectrum of compound **4b**.

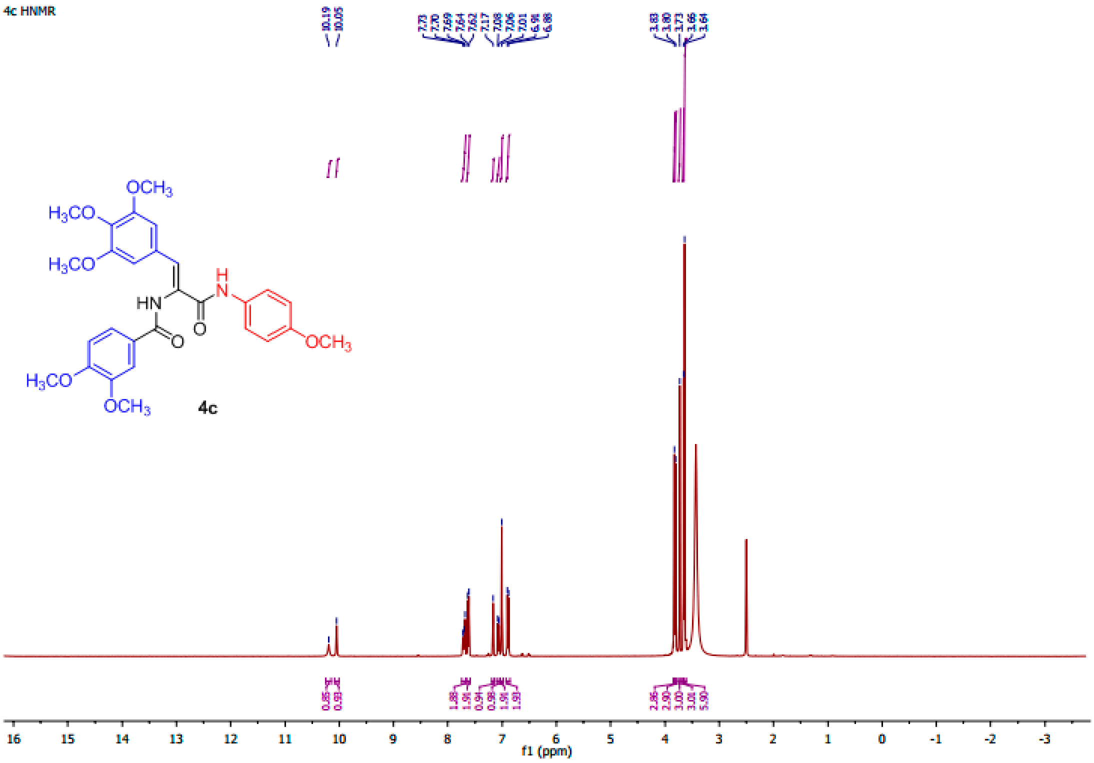

Figure S15. ¹H-NMR spectrum of compound **4c**.

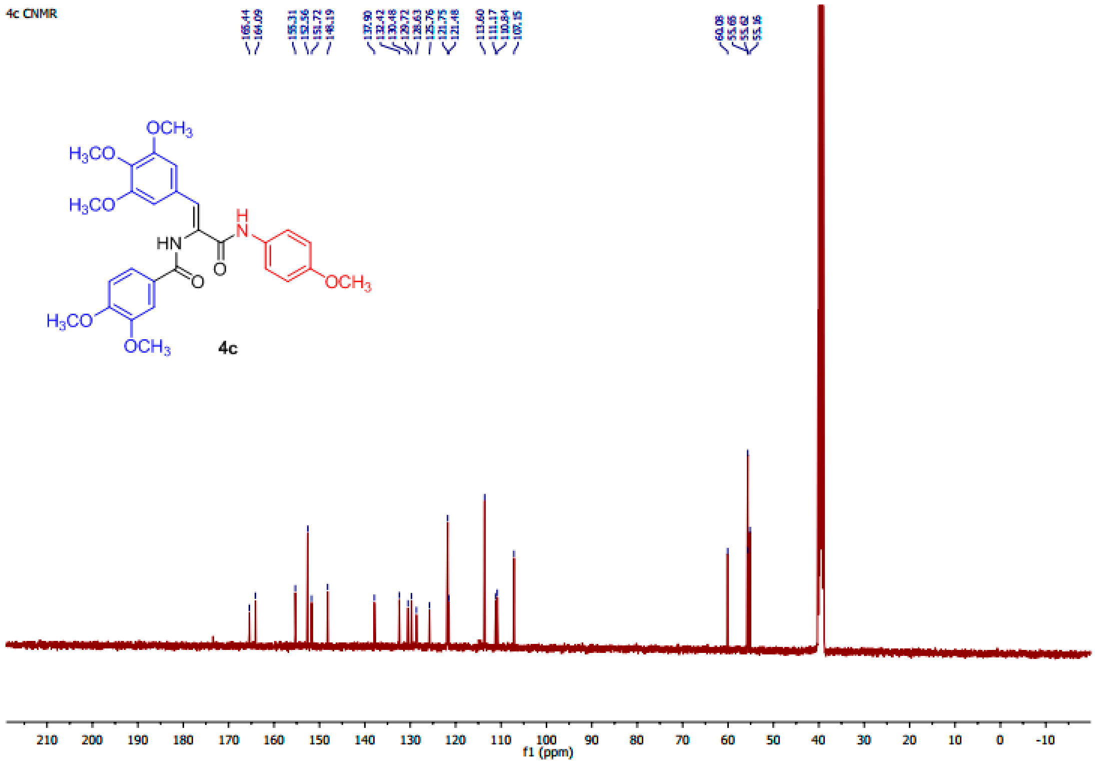

Figure S16. ¹³C-NMR spectrum of compound **4c**.

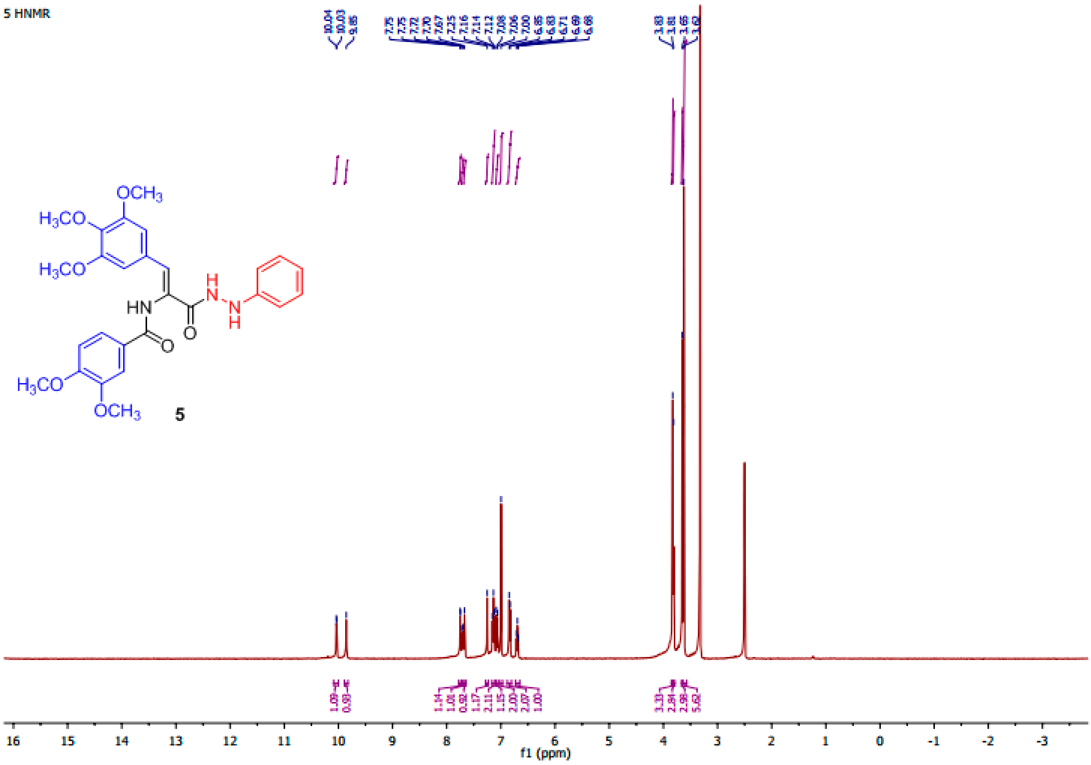

Figure S17. ^{1}H-NMR spectrum of compound **5**.

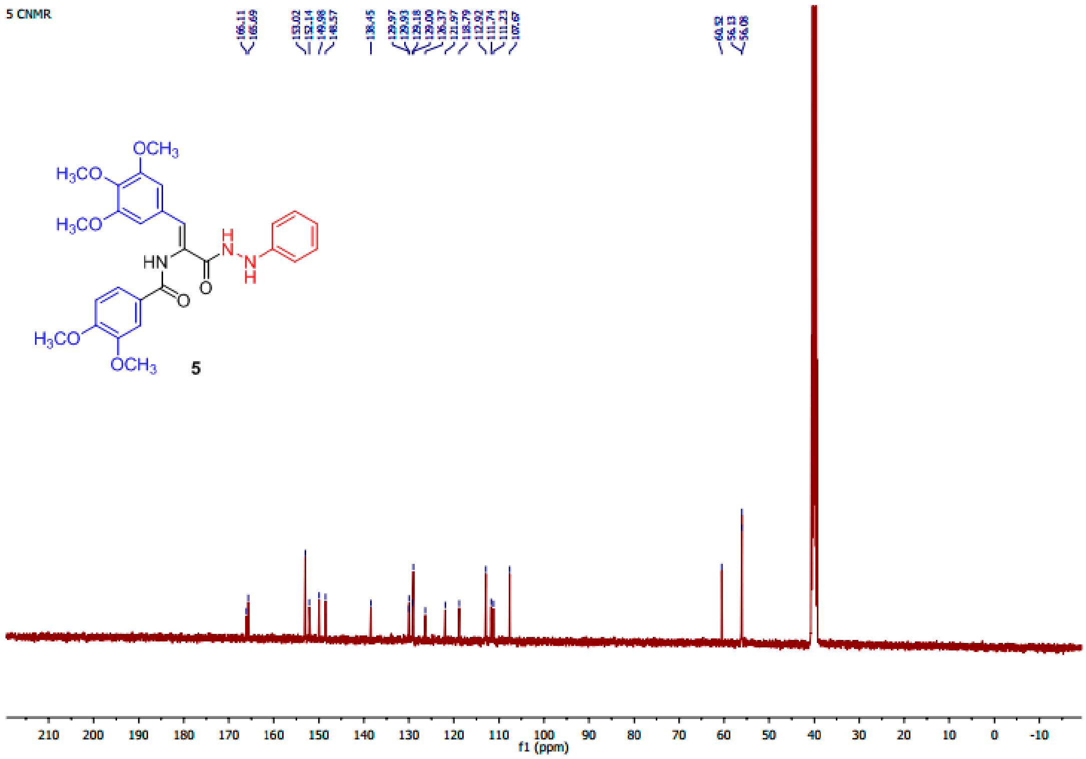

Figure S18. ^{13}C-NMR spectrum of compound **5**.

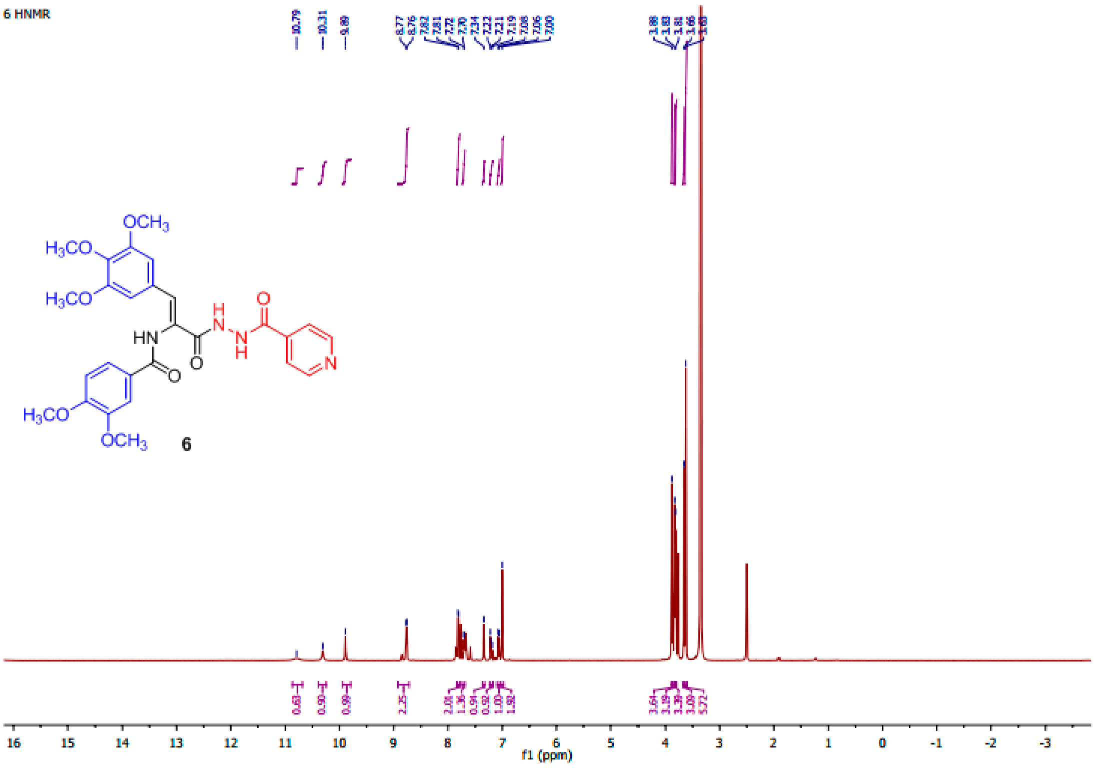

Figure S19. ^{1}H-NMR spectrum of compound **6**.

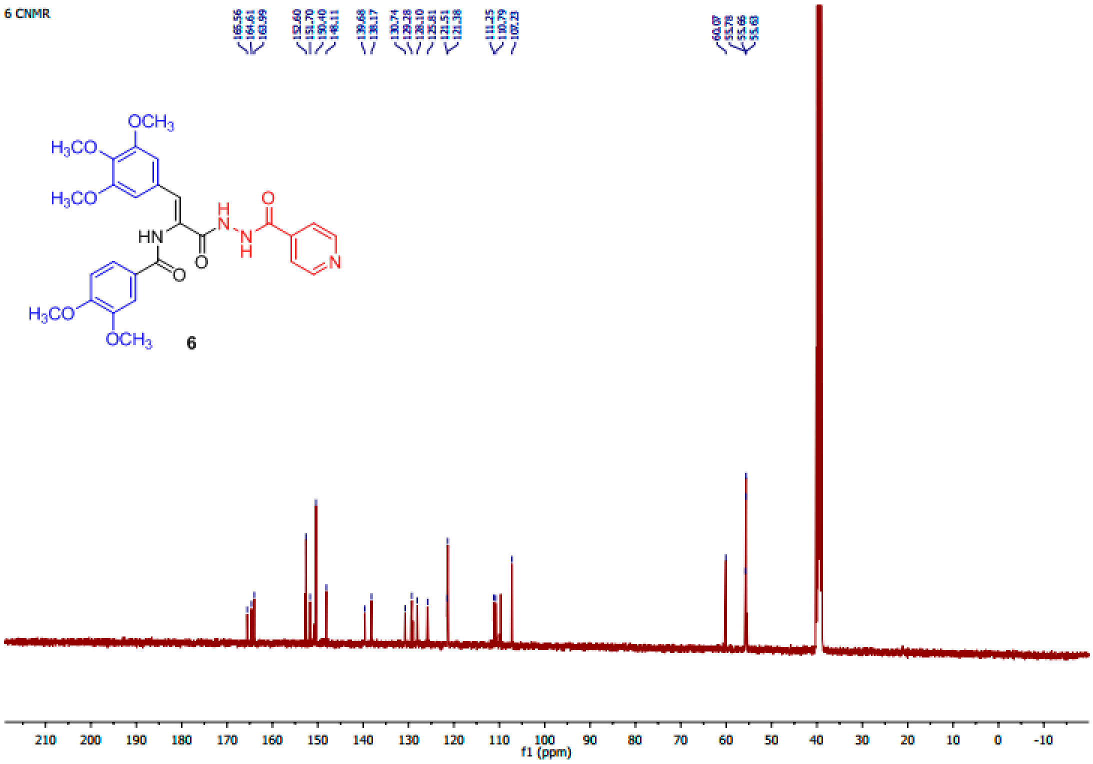

Figure S20. ^{13}C-NMR spectrum of compound **6**.

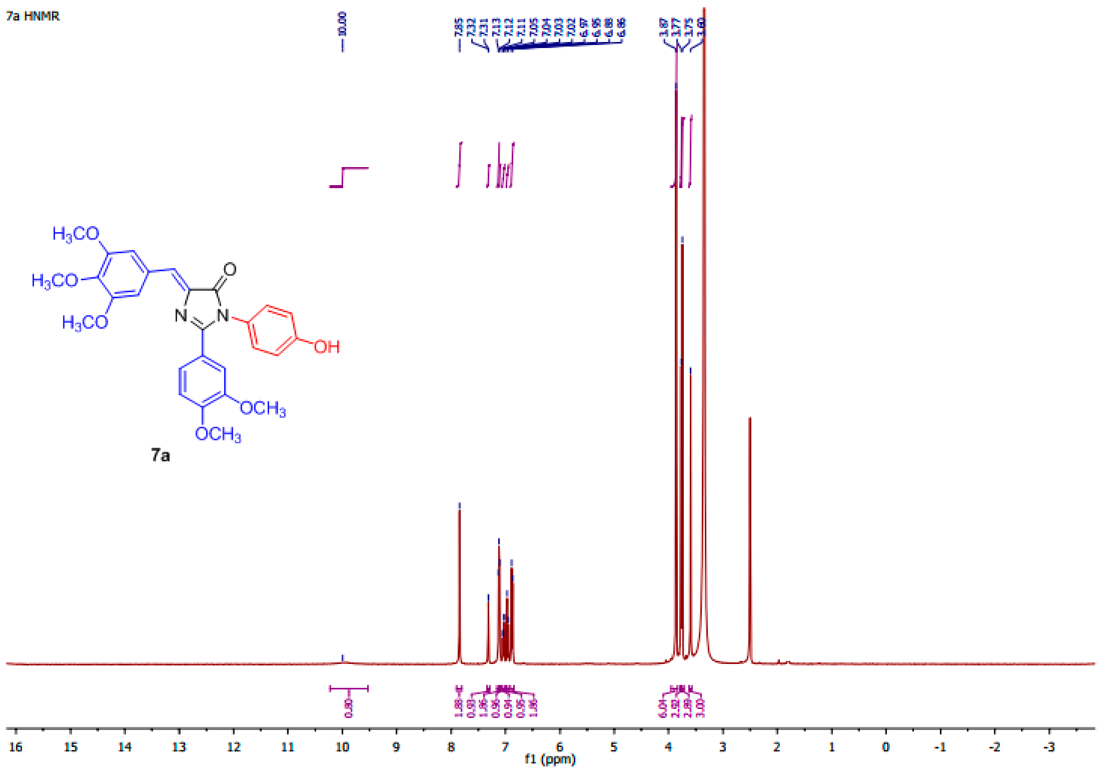

Figure S21. ^{1}H-NMR spectrum of compound **7a**.

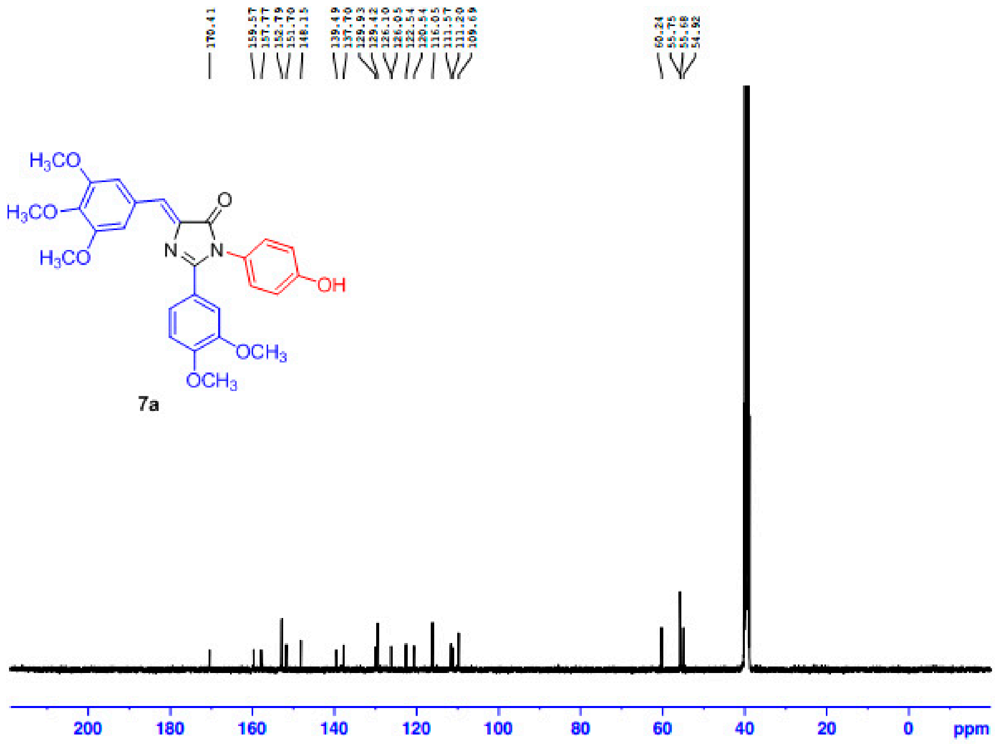

Figure S22. ^{13}C-NMR spectrum of compound **7a**.

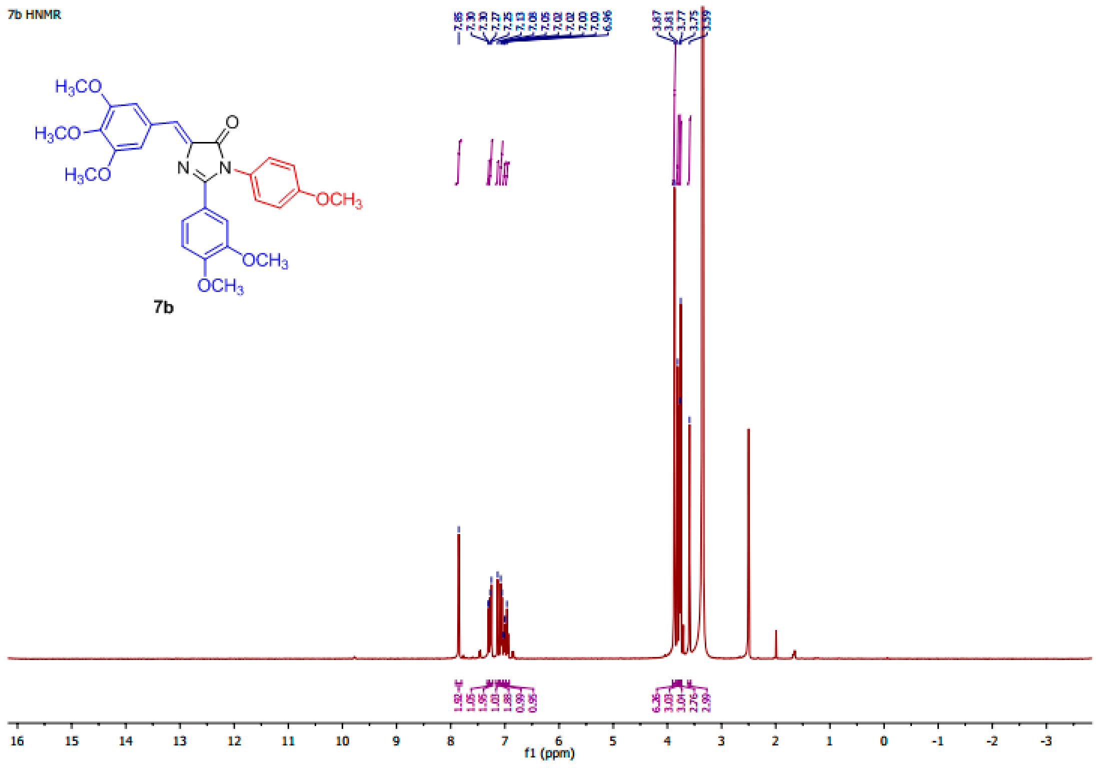

Figure S23. ^{1}H-NMR spectrum of compound **7b**.

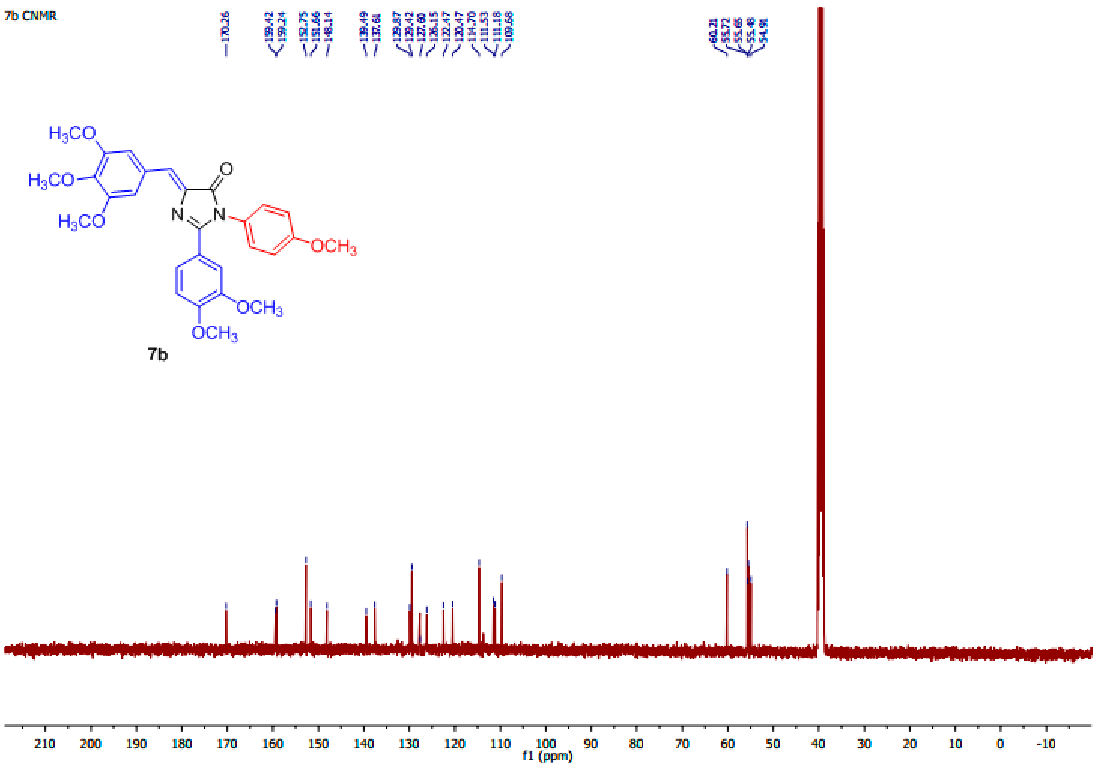

Figure S24. ^{13}C-NMR spectrum of compound **7b**.

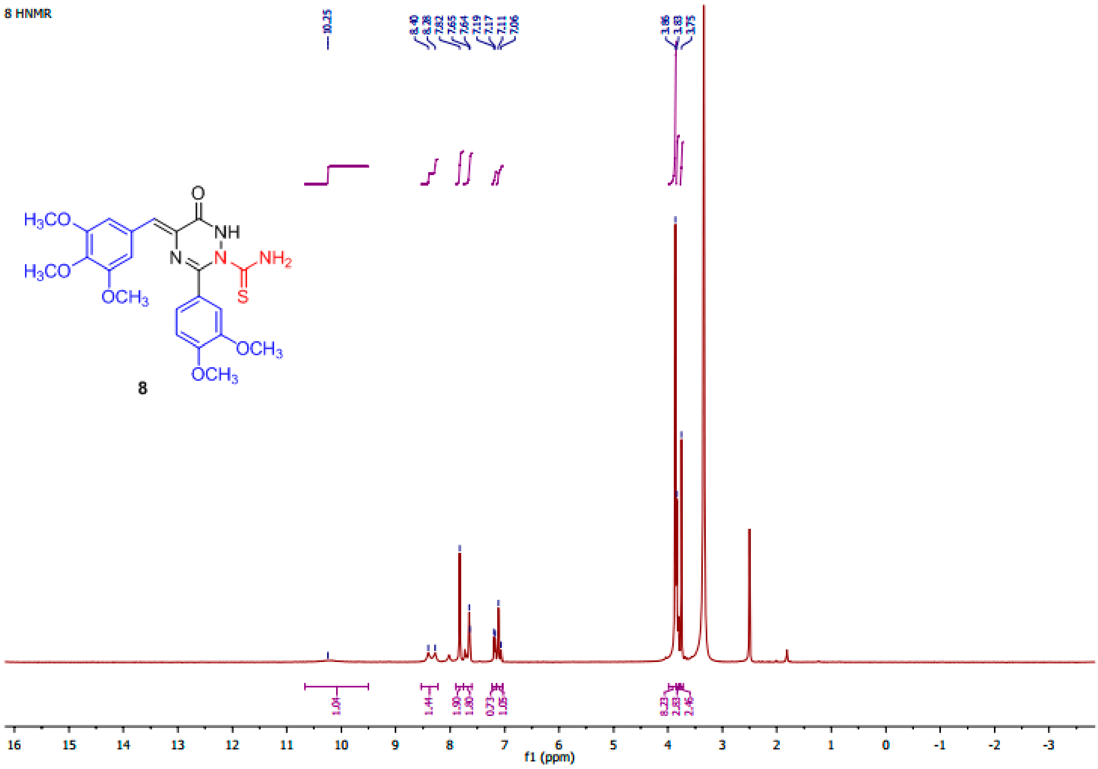

Figure S25. ^{1}H-NMR spectrum of compound **8**.

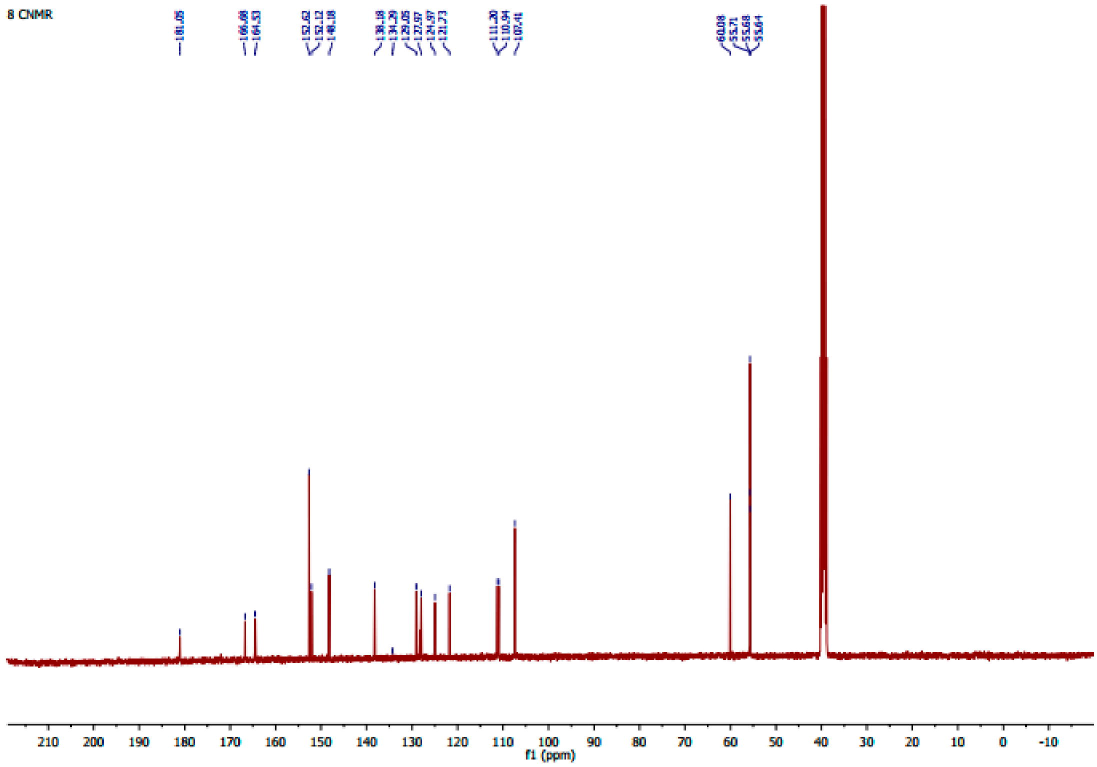

Figure S26. ^{13}C-NMR spectrum of compound **8**.

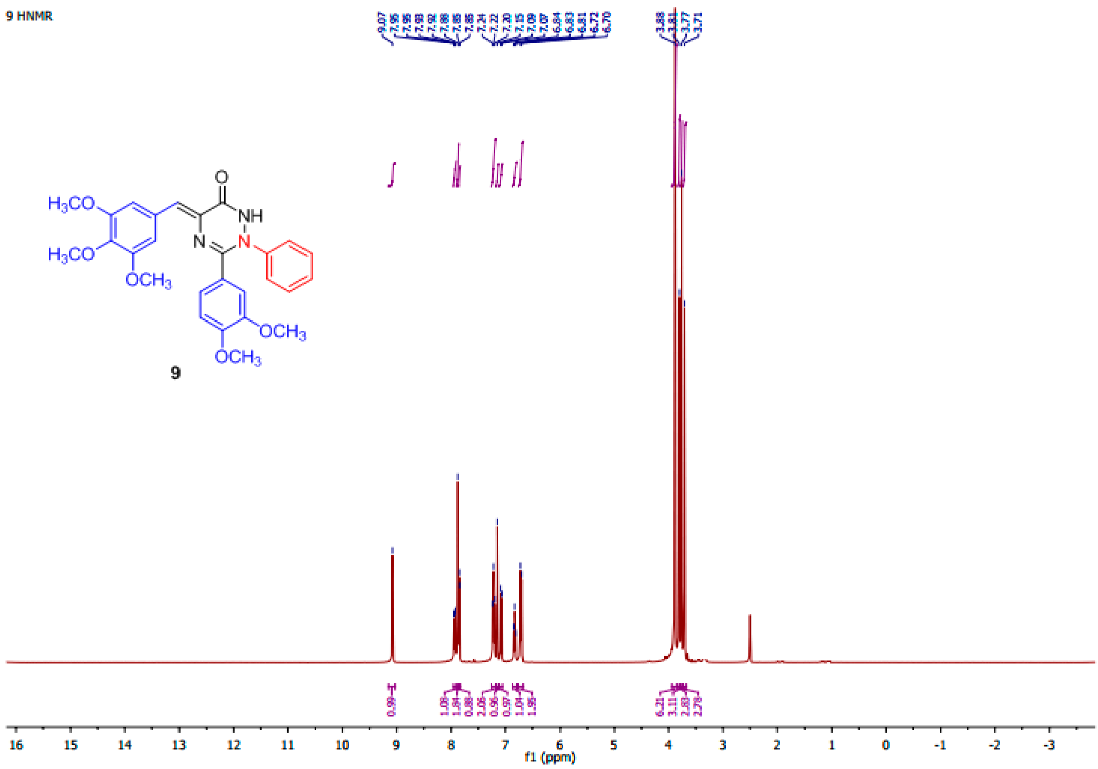

Figure S27. ^{1}H-NMR spectrum of compound **9**.

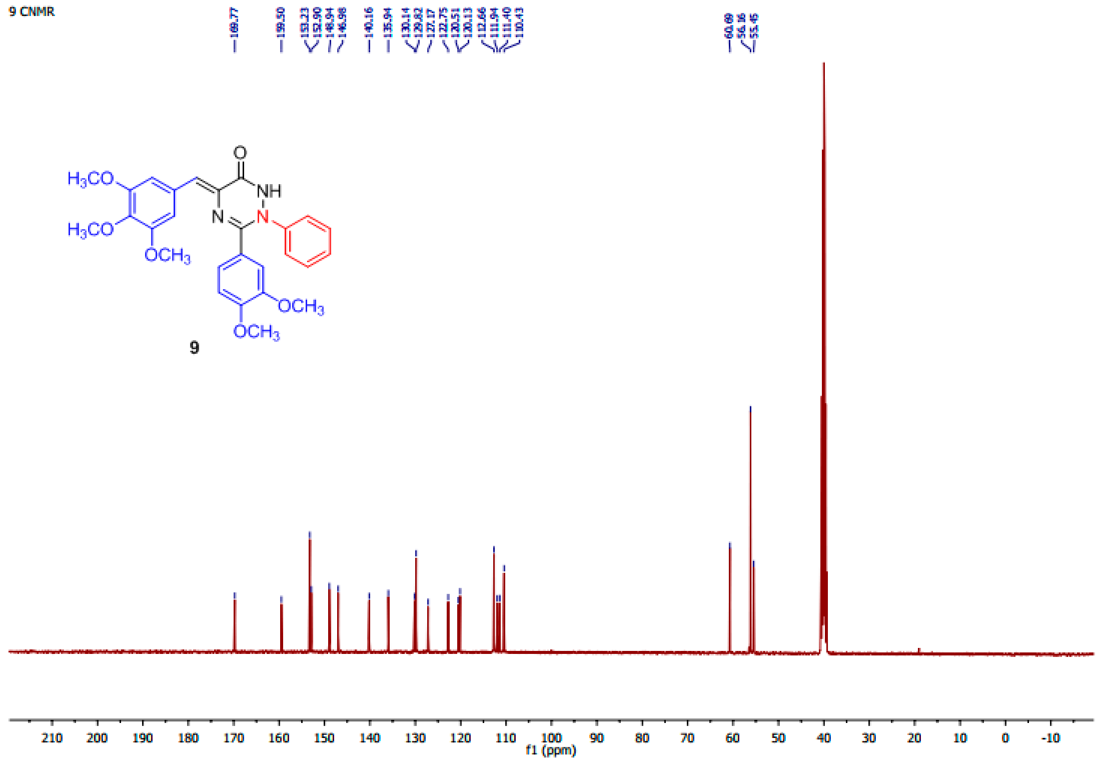

Figure S28. ^{13}C-NMR spectrum of compound **9**.

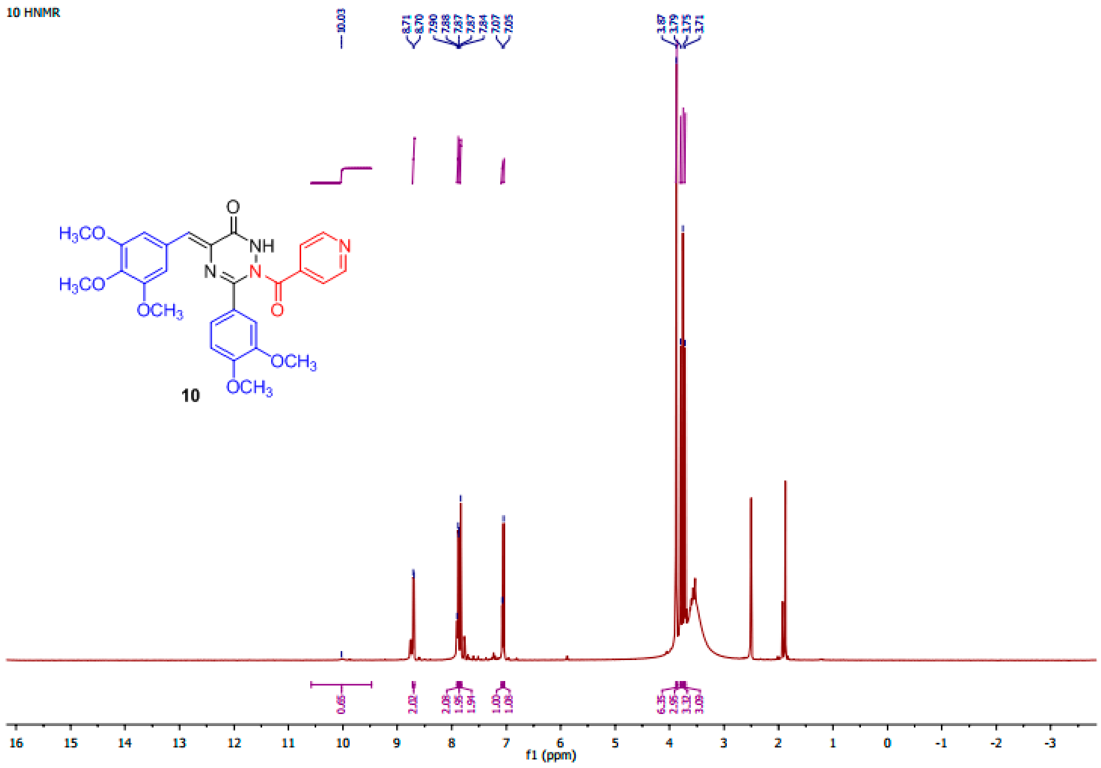

Figure S29. ^{1}H-NMR spectrum of compound **10**.

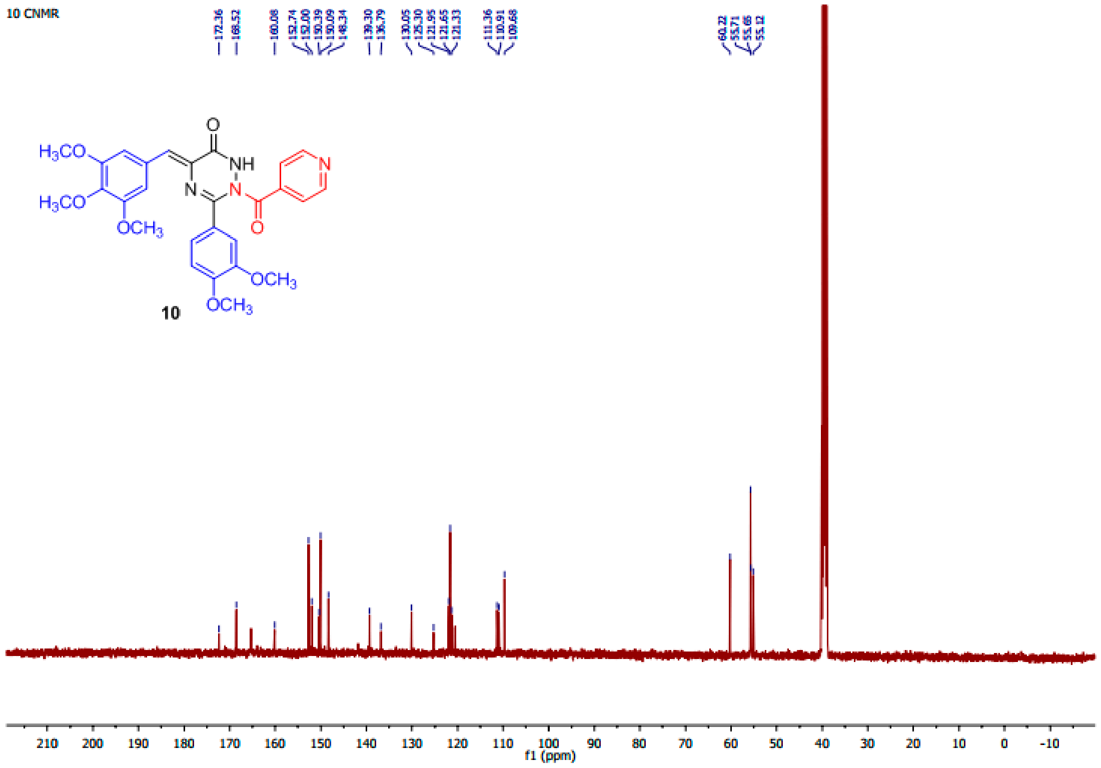

Figure S30. ^{13}C-NMR spectrum of compound **10**.

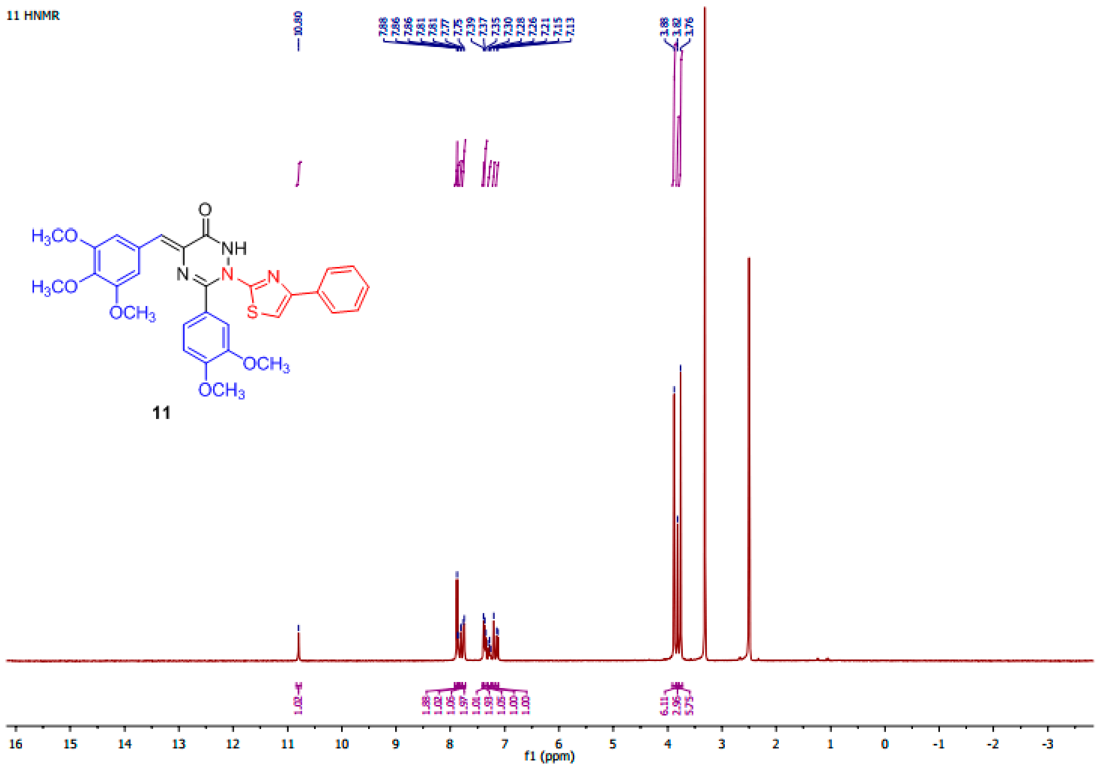

Figure S31. ^{1}H-NMR spectrum of compound **11**.

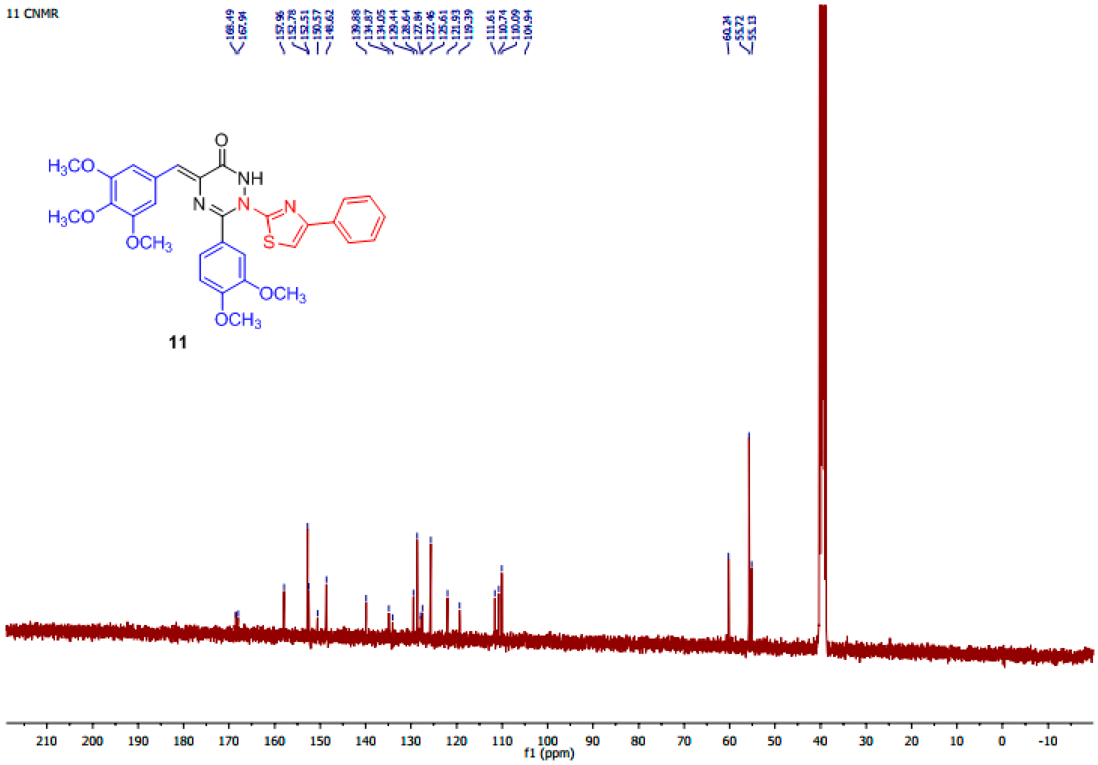

Figure S32. ^{13}C-NMR spectrum of compound **11**.

3.3. Biological studies

3.3.1. Cytotoxic activity evaluation

To measure the cytotoxic activity of the synthesized derivatives **2a-11** in hepatocellular carcinoma (HepG2) cell line (ATCC Cat No. HB-8065) as well as normal human liver cell line HL-7702 (ATCC Cat. No. 77402), cell viability assay was assessed using MTT assay method. Cells at density of 1 x 10^4 were seeded in a 96-well plate at 37 ^{o}C for 24 h under 5% CO_2. After incubation, the cells were treated with different concentrations of the test compounds **2a-11** and incubated for 24 h, then 20 µl of MTT solution at 5 mg/mL was applied and incubated for 4 h at 37 °C. Dimethyl sulphoxide (DMSO) in volume of 100 µl was added to each well to dissolve the purple formazan that had formed. The color intensity of the formazan product, which represents the growth condition of the cells, is quantified by using an ELISA plate reader (EXL 800, USA) at 570 nm absorbance. The experimental conditions were carried out with at least three replicates, and the experiments were repeated at least three times.

3.3.2. Tubulin inhibitions Assay

Compounds **9, 10** and **11** were evaluated for their tubulin inhibitory activity levels according to the manufacturer's instructions using # abcam Human Beta-tubulin simplestep ELISA Kit ab245722.

3.3.3. Cell cycle analysis of compound 9

Cell cycle analysis in HepG2 cells was investigated using fluorescent Annexin V-FITC/ PI detection kit (*BioVision* EZCell™ Cell Cycle Analysis Kit Catalog #K920) by flow cytometry assay. HepG2 cells at a density of 2 × 10^5 per well were harvested and washed twice in PBS. After that, the cells were incubated at 37 °C and 5% CO_2. The medium was incubated with the tested compound **9** at its IC_{50} (µM) for 48 h, washed twice in PBS, fixed with 70% ethanol, rinsed again with PBS. Afterward, medium was stained with DNA fluorochrome PI for 15 min at 37 °C. The samples were immediately analyzed using Facs Calibur flow cytometer (Becton and Dickinson, Heidelberg, Germany).

3.3.4. Apoptosis assay for compound 9

Apoptosis in HepG2 cells was investigated using fluorescent Annexin V-FITC/ PI detection kit (*BioVision* Annexin V-FITC Apoptosis Detection Kit, Catalog #: K101) by flow cytometry assay. HepG2 cells at a density of 2 × 10^5 per well were treated with compound **9** at its IC_{50} (µM) for 48 h, then the cells were harvested and stained with Annexin V-FITC/ PI dye for 15 min in the dark at 37 °C. The samples were immediately analyzed using *FACS Calibur* flow cytometer (Becton and Dickinson, Heidelberg, Germany).

3.3.5. Measurement of mitochondrial membrane potential (MMP) for compound 9

MMP was measured by FACS analysis using abcam ab113852TMRE Mitochondrial Membrane Potential Assay Kit with DiOC2(3) staining using MitoProbe™ DiOC2(3) Assay Kit for Flow Cytometry (M34150). After treatment with compound **9** at its IC_{50} concentration, cells were stained with DiOC2(3) dye for 30 min in the incubator, then harvested and washed with PBS. DiOC2(3)-stained cells were represented with Annexin binding buffer and incubated at 37 ^{o}C for 15 min. The samples were immediately analyzed using cell Quest software.

3.3.6. Effect on p53, Bax and Bcl-2

p53, Bax and Bcl2 enzyme activities in HepG2 cells were detected in the presence of compound **9** at its IC_{50} concentration (µM). The levels of the tumor suppressor gene p53, apoptotic markers Bax as well as the anti-apoptotic marker Bcl-2 were assessed using p53 ELISA kit, Human Bax ELISA kit and Bcl-2 Elisa kit. The procedure of the used kits was done

according to the manufacturer's instructions. Briefly, cell lysates were prepared from control and HepG2 cells (2.5 x 10^5/mL) treated with IC_{50} concentration of compound **9**. Then, equal amounts of cell lysates were loaded and then probed with specific antibodies. The samples were measured at 450 nm in ROBONEK P2000 ELISA reader. Analysis was confirmed with three different sets of extracts. All experiments were done in triplicates.

3.3.7. Molecular docking study

Molecular docking study was performed using MOE software program (MOE 2009.10). The tubulin crystal structure (PDB code: 5LYJ) was obtained from protein data bank. All molecular docking calculations and docking studies into the active site of 5LYJ of compound **9** were carried out using Molecular Operating Environment (MOE 2009.10) software. Energy minimizations were performed with a root mean standard deviation (RMSD) gradient of 0.05 kcal/mol^{-1} $Å^{-1}$ with an MMFF94X force field and the partial charges were calculated. The 3D structures of the ligands were built using MOE and subjected to the following procedure: (i) 3D protonation of the structure; (ii) hide of the hydrogen (iii) selecting the least energetic conformer. Moreover, the target 5LYJ was prepared for docking studies as follows: (i) water molecules were discarded; (ii) hydrogen atoms were added to the enzyme; (iii) MOE alpha site finder was used for the active site search; (iv) docking of the targeted ligands.
